# DNA-directed electrochemiluminescence nanosphere with electrocatalysis-enhanced microfluidic arrays for rapid multibacterial detection

**DOI:** 10.1126/sciadv.ady3070

**Published:** 2025-12-12

**Authors:** Chengli Zhang, Xiaolong Guo, Judun Zheng, Yi Feng, Wenjie Wu, Lunjing Liu, Jiang Xiao, Qingxian Li, Huiyi Yang, Jingru Wang, Jiajian Zhou, Yu Fu, Yuhui Liao

**Affiliations:** ^1^ Dermatology Hospital, Southern Medical University, Guangzhou 510091, P.R. China.; ^2^ Institute for Engineering Medicine, Kunming Medical University, Kunming 650500, P.R. China.; ^3^ Department of Laboratory Medicine, Zigong Fourth People’s Hospital, Zigong 643000, P.R. China.; ^4^ Zhongshan City Prople’s Hospital, Zhongshan 528400, P.R. China.; ^5^ Ningxia Medical University, Yinchuan 750004, P.R. China.

## Abstract

Rapid detection of multibacterial pathogens is crucial for accelerating the diagnosis and treatment of bacterial infections. We propose a rapid and efficient electrochemiluminescence (ECL) sensor for the synchronous detection of multiple bacterial pathogens, including *Staphylococcus aureus*, *Klebsiella pneumoniae*, *Pseudomonas aeruginosa*, and methicillin-resistant *S. aureus*. This homogeneous sensor is based on a self-assembled DNA nanosphere loaded with tetrakis (4-carboxyphenyl) porphyrin (TCPP). The sensor operates in an “off-on” mode, in which bacterium-aptamer binding triggers a conformational change in the DNA nanosphere, releasing TCPP and generating an enhanced ECL signal. The inclusion of cerium nanoparticles boosts signal intensity through electrocatalytic reactions, improving sensitivity with a detection limit of ≤100 colony-forming units per milliliter. Integrated with a microfluidic chip, the system enables multibacterial detection in just 45 minutes. Bacterial quantification in clinical samples strongly correlates with digital polymerase chain reaction results. This approach provides a rapid, specific, and efficient diagnostic tool for bacterial infections with great potential for point-of-care applications in clinical settings.

## Teaser

An advanced ECL sensor has been developed for multibacterial detection, offering point-of-care diagnostics with high sensitivity.

## INTRODUCTION

Bacterial infections are a leading cause of illness and death worldwide (*1*). Common pathogens, including *Staphylococcus aureus* (*S. aureus*), methicillin-resistant *S. aureus* (MRSA), *Klebsiella pneumoniae* (*K. pneumoniae*), and *Pseudomonas aeruginosa* (*P. aeruginosa*) (*1*, *2*), cause localized tissue damage and trigger a systemic inflammatory response, resulting in multiorgan failure and, in severe cases, death (*3*–*6*). The clinical features of bacterial infections often overlap with those of other diseases (*7*, *8*), and different bacterial species can cause similar symptoms (*9*), leading to a challenging clinical diagnosis. Bacterial infections are typically diagnosed using bacterial culture methods (*6*, *10*), nucleic acid testing (*11*, *12*), and time-of-flight mass spectrometry (MALDI-TOF MS) (*13*). However, these methods are cumbersome, time-consuming, and may delay treatment. Furthermore, several bacteria may work together to cause complex infections (*14*). For example, *P. aeruginosa* can enhance the virulence of *S. aureus*, leading to severe inflammation and delayed healing (*15*). Mixed infections of *P. aeruginosa* and *K. pneumoniae*, both biofilm forming and antibiotic resistant, can complicate treatment (*16*, *17*). Single bacterial tests often fail to provide a comprehensive understanding of an infection. Therefore, a rapid method for characterizing multiple bacteria is urgently required to facilitate timely and accurate diagnoses and more effective treatments.

Various advanced techniques, such as three-dimensional hierarchical nanotopography (*18*), CRISPR-Cas13a–based electrochemical biosensors (*19*), droplet encoding-pairing enabled multiplexed digital loop-mediated isothermal amplification (*20*), and metagenomic next-generation sequencing (*21*), have been used to detect multiple pathogens accurately. However, these approaches have limitations, such as operational complexity, high detection costs, and lengthy processing times. Recently, electrochemiluminescence (ECL) technology has made considerable progress in improving operational procedures, controllability, cost-effectiveness, and sensitivity, making it a promising approach for bioanalysis and medical diagnostics (*22*). ECL can be used for simultaneous multitarget detection, which is particularly beneficial for identifying multiple bacterial pathogens in a single assay. Now, most multitarget ECL sensors rely on potential-resolved systems that use multiple luminophores (*23*, *24*). However, this strategy depends on the development of advanced luminophores. Overlapping signals from different ECL luminophores can compromise the accuracy of simultaneous multitarget detection. Additionally, conventional ECL assays are heterogeneous methods, which can limit detection efficiency.

To address these challenges, this study integrates DNA nano-self-assembly technology into an ECL system to achieve rapid and homogeneous multibacterial detection. The self-assembled DNA nanostructures are typically formed by folding long single-stranded DNA scaffolds with short “stapled” strands, enabling precise control over their shape and function (*25*, *26*). For example, dynamic DNA nanostructures, designed by incorporating aptamers, complementary sequences, or other responsive elements, can undergo structural transitions, enabling the controllable release of functional components such as fluorescent dyes, small interfering RNAs, immunostimulatory sequences, and anticancer drugs (*27*–*29*). This makes them ideal for drug delivery, molecular imaging, and biosensing applications. Inspired by this, we synthesized a functional “closed (off)–open (on)” DNA helical nanosphere based on bacterial aptamers and incorporated tetrakis (4-carboxyphenyl) porphyrin (TCPP) as an ECL luminophore into the nanosphere, forming a DNA helical nanosphere–TCPP complex (DNA ECL nanosphere). The bacteria-specific aptamer acts as interlocking chains, ensuring that the nanosphere remains enclosed. Upon stimulation by a specific bacterium (key), the aptamer undergoes a conformational change, causing the low-conductivity DNA ECL nanosphere to open and release TCPP. This transition triggers an immediate change in the ECL signal, enabling rapid bacterial detection.

In this study, four bacterial diagnostic reagents were synthesized using four aptamers as recognition probes to enable multibacterial detection, each targeting a specific bacterial species. The target bacteria included *S. aureus*, MRSA, *K. pneumoniae*, and *P. aeruginosa*. To enhance detection efficiency, we integrated microfluidic technology into manual spiking assays, incorporating key functions such as reagent and sample loading, mixing, and incubation, thereby streamlining the process and reducing assay time.

Furthermore, ECL signal amplification, crucial for enhancing the assay sensitivity, was achieved by incorporating ceria nanoparticles (NanoCe). Cerium ions have unique electrocatalytic properties (*30*, *31*), especially the Ce^4+^/Ce^3+^ redox pair (*32*, *33*), which can effectively promote electron transfer during in the ECL reaction. The synthesized NanoCe acted as an accelerator in the S_2_O_8_^2−^-TCPP ECL system, which synergized with coreactant S_2_O_8_^2−^ through an electrocatalytic reaction. This promoted the generation of more coreactant radicals and amplified the ECL signal, improving the overall performance and sensitivity of the sensor.

**Fig. 1.**
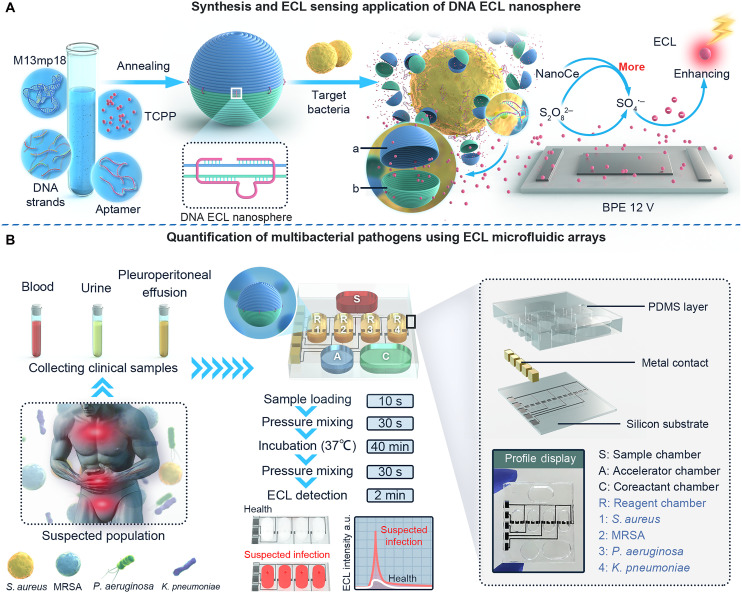
Principle of the multibacterial ECL microfluidic arrays. (**A**) Schematic illustrating the synthesis and ECL sensing application of the DNA ECL nanospheres as diagnostic reagents. (**B**) Schematic diagram showing the multiplex quantification of bacterial pathogens using ECL microfluidic arrays. a.u., arbitrary units.

Last, a homogeneous ECL microfluidic array was constructed by combining the DNA ECL nanosphere, the NanoCe-enhanced TCPP-S_2_O_8_^2−^ ECL system, and microfluidic technology. This assay can efficiently detect multibacterial pathogens (Fig. 1) using four reagents that were preloaded into the microfluidic chip. Consequently, the detection can be completed within 45 min without any special operation, with a limit of detection (LOD) of ≤100 colony-forming units (CFU)/ml. The ECL microfluidic array also accurately quantified *S. aureus*, MRSA, *P. aeruginosa*, and *K. pneumoniae* from various clinical samples, including urine, blood, and pleural and abdominal fluids, with high consistency. This ECL microfluidic arrays offers several advantages over traditional methods, such as shorter detection time, ease of use, and low cost, demonstrating its potential for the clinical diagnosis of multiple pathogens. Moreover, the platform can potentially be applied to other target-aptamer recognition modalities.

## RESULTS

### Principle of the multibacterial ECL microfluidic arrays

Figure 1 illustrates the fabrication process of the homogeneous multibacterial ECL microfluidic arrays. The DNA ECL nanosphere was first synthesized as diagnostic reagent using a one-step method, including 166 fixed and 5 variable short DNA strands, a variable aptamer strand, a M13mp18 single-stranded DNA, and the TCPP luminophore (Fig. 1A). The five variable short DNA strands used in DNA ECL nanosphere were based on the aptamer sequence, with each strand differing across various bacteria. This design ensured that the sequences could reliably bind to the aptamer and other strands through base complementarity. By altering the aptamer sequences and five variable short DNA strands during synthesis, diagnostic reagents could be synthesized to individually recognize *S. aureus*, MRSA, *P. aeruginosa*, and *K. pneumoniae* (please refer to Methods and Methods and table S1 for the DNA sequences). The DNA helical nanosphere was composed of two hemispheres, “a hemisphere” and “b hemisphere” that could exist in “closed” or “open” conformations in a lock (aptamer)–key (target) system. Each hemisphere was modified with single-stranded DNA (ssDNA) overhangs at five positions along the equator. Each protruding ssDNA could not hybridize with the other strand at the same position. The aptamer strand switched to a closed conformation by hybridizing with two ssDNA overhangs. This closed structure could be “opened” when stimulated by a target that releases the aptamer. In the presence of the target bacteria, the aptamer specifically bound to the bacteria and underwent a conformational change that switched the DNA nanosphere from a closed to an open state. Consequently, TCPP was released from the low-conducting DNA nanosphere and mixed with S_2_O_8_^2−^ and NanoCe, resulting in an enhanced ECL signal that was detected using a bipolar electrode at 12 V.

Microfluidic technology was integrated into the ECL system to automate the assay, which was designed for the rapid and sensitive detection of four distinct targets. As illustrated in Fig. 1B, the microfluidic chip consists of a silicon substrate, a polydimethylsiloxane (PDMS) layer, and metal contact. The carbon working and driving electrodes were printed on a silicon substrate to provide an optimal voltage and activate the ECL reaction. Four metallic contacts were inserted to connect the carbon electrode to the detection apparatus. The PDMS layer consisted of four reagent chambers (Reagent*_S. aureus_*, Reagent_MRSA_, Reagent*_P. aeruginosa_*, and Reagent*_K. pneumoniae_*), two coreactant chambers (Accelerator_NanoCe_/Coreactant_S2O82−_), and a sample chamber, which were used for reagent storage (reaction incubation), coreactant storage, and sample loading, respectively.

Before sample analysis, the detection reagents were preloaded into the designated chambers. Initially, a sample was added to the sample chamber of the microfluidic chip and pressed into the four reagent chambers using a stepper motor, which dispensed the sample at a controlled rate. The microfluidic chip was then heated to 37°C and maintained at this temperature for 40 min. The S_2_O_8_^2−^ and NanoCe were introduced into the reagent chambers via a stepper motor. Last, the microfluidic chip was inserted into the auxiliary testing equipment for ECL detection. The positive samples exhibited an enhanced ECL signal.

Unlike the traditional “lock and key” model, this assay does not require a wash step. In addition to its high specificity, this assay is user-friendly and has a short detection time. Furthermore, the microfluidic PDMS layer is prone to embedding small molecules inside, while heterogeneous assays require a wash step to eliminate nonspecific signal molecules, making false positive results practically unavoidable. However, this homogeneous detection system can obtain specific signals without washing, thus circumventing the problem of nonspecific retention of ECL molecules due to incomplete washing.

### Characterization of the DNA helical nanosphere and the DNA ECL nanosphere

**Fig. 2.**
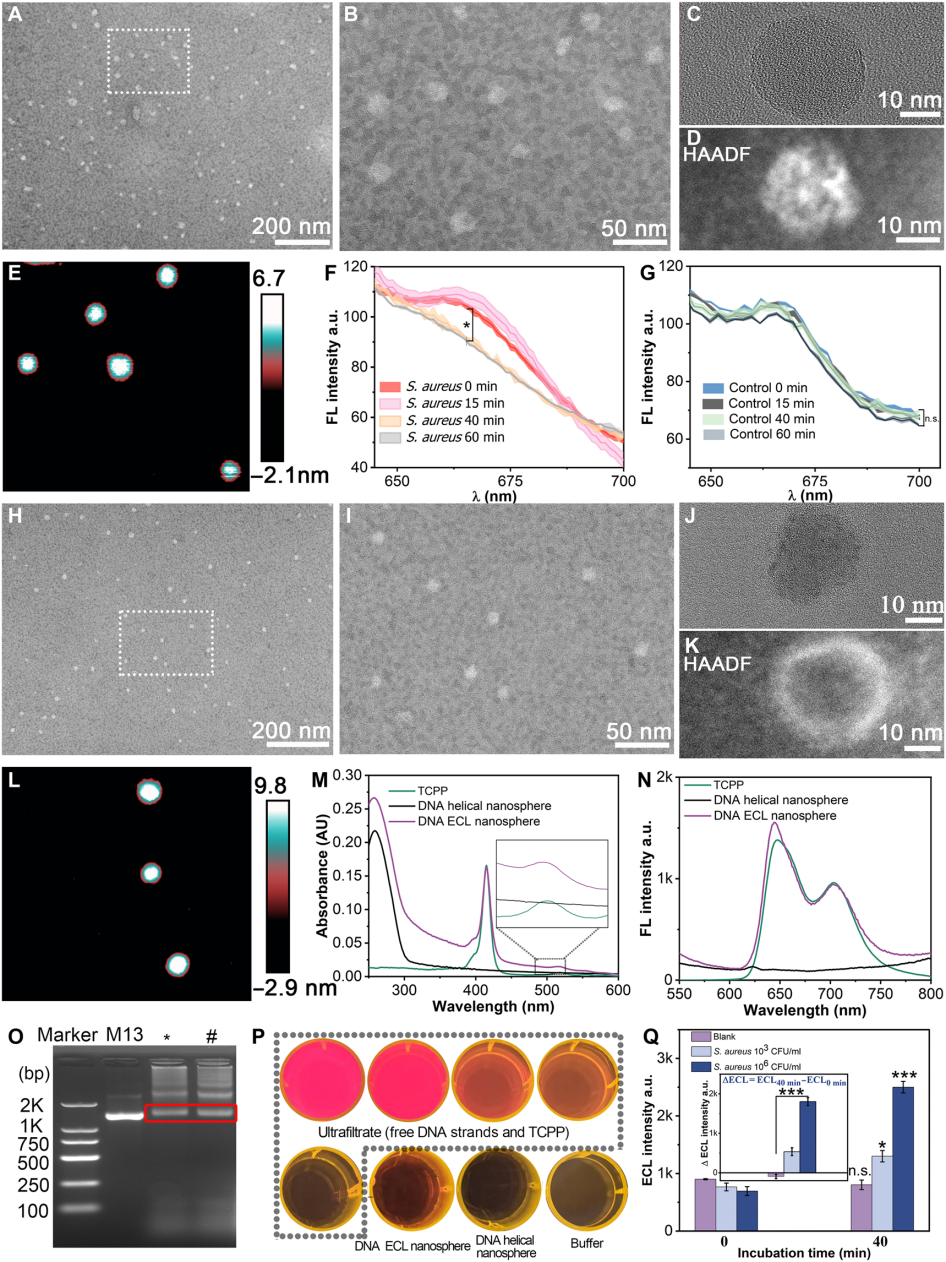
Characterization of DNA helical nanosphere and DNA ECL nanosphere. (**A** and **B**) Transmission electron microscopy (TEM) images of DNA helical nanospheres using the negative staining technique. (**C**) TEM image of DNA helical nanospheres. (**D**) High-angle annular dark-field (HAADF)–TEM image of DNA helical nanospheres. (**E**) Atomic force microscopy (AFM) image of DNA helical nanospheres. (**F** and **G**) Verification of the transition of DNA helical nanosphere from the closed to the open state. The light-shaded area indicates the 95% confidence interval of the data. (**H** and **I**) TEM images of DNA ECL nanospheres using the negative staining technique. (**J**) TEM image of DNA ECL nanospheres. (**K**) HAADF-TEM image of DNA ECL nanospheres. (**L**) AFM image of DNA ECL nanospheres. (**M**) Ultraviolet-visible (UV-vis) absorption spectra. (**N**) Fluorescence spectra. (**O**) Electropherogram (agarose gel electrophoresis), M13, M13mp18; *, DNA helical nanosphere; and #, DNA ECL nanosphere. bp, base pairs. (**P**) Fluorescent photograph. (**Q**) ECL results for the release of TCPP from DNA ECL nanosphere. The experiments were repeated for three times (*n* = 3) and data were presented as means ± SD. Statistical analysis comparing 0 min versus 40 min for the blank, 10^3^ CFU/ml, and 10^6^ CFU/ml groups. *P < 0.05; ***P < 0.001; n.s., not significant. AU, absorbance units. a.u., arbitrary units.

Transmission electron microscopy (TEM) and atomic force microscopy (AFM) were used to characterize the size and morphology of the as-prepared DNA helical nanosphere (Fig. 2). A uniform population of DNA nanostructures was subjected to negative staining and observed via TEM (Fig. 2, A and B). High-resolution TEM and high-angle annular dark-field (HAADF) results indicated that the average diameter of DNA helical nanospheres was ~20 nm (Fig. 2, C and D). The AFM images showed islands ~10 nm in height (Fig. 2E), consistent with the previous report (*27*).

Using *S. aureus* as the target model, we incorporated an aptamer sequence that specifically recognizes *S. aureus* in the lock strand to verify the response of the DNA helical nanosphere to the target. This *S. aureus*–specific DNA helical nanosphere was used in subsequent experiments. The transition from the “off” to the “on” state was investigated using fluorescence resonance energy transfer (FRET). The fluorescent dyes (Cy3 and Cy5) were conjugated to the 3′ and 5′ ends of the *S. aureus* aptamer strand, respectively. The closed conformation allowed both fluorophores to be in close proximity, generating the FRET signal, while the open conformation did not. The emission peak of Cy5 was observed, indicating that the DNA helical nanosphere was in the closed conformation. After adding *S. aureus*, the Cy5 fluorescence signals decreased in a time-dependent manner (Fig. 2F) and were minimal after 40 min of incubation. No statistical changes were observed in the fluorescence signal of the control group (Fig. 2G). These results indicated that the presence of *S. aureus* successfully triggered the opening of the DNA helical nanosphere due to the specificity of the *S. aureus* aptamer to its target.

On the basis of these findings, we further synthesized the DNA ECL nanosphere, the primary reagent of this detection strategy. TEM, HAADF, and AFM results showed that the structure and morphology of the DNA ECL nanospheres remained unchanged even after TCPP was loaded into the nanosphere cavity (Fig. 2, H to L). Consistent with the TEM results, the dynamic light scattering data showed a uniform size distribution peak (fig. S1). Furthermore, the DNA helical nanosphere and DNA ECL nanosphere showed similar mobility when electrophoresed on a 1.5% agarose gel, consistent with previous reports (Fig. 2O) (*27*). The nanosphere remained stable after being stored for 2 hours at 37°C (fig. S2, A and B). Moreover, the ζ-potentials of TCPP, the DNA helical nanosphere, and the DNA ECL nanosphere were −47.5, −31.5, and −35 mV, respectively, indicating that the surface charge of the nanosphere remained largely unaffected after TCPP loading (fig. S3).

As TCPP loading is a critical step, the assembly of the TCPP and the DNA helical nanosphere were characterized using ultraviolet-visible (UV-vis) absorption spectra, fluorescence spectra, fluorescence photo, and confocal laser scanning microscopy (CLSM). TCPP displayed a strong absorption peak at 415 nm and a weak one at 520 nm (*34*). The DNA helical nanosphere exhibited the characteristic absorption peak of DNA molecules at 260 nm (*35*). Upon TCPP loading onto the DNA helical nanosphere, the resulting DNA ECL nanosphere showed the characteristic absorption peaks of DNA and TCPP (Fig. 2M). When excited at 415 nm, TCPP exhibited emission peaks at 640 and 700 nm. The DNA helical nanosphere, which showed no fluorescence on their own, exhibited the same emission peaks after TCPP loading (Fig. 2N). In parallel, fluorescence from the DNA helical nanospheres and the DNA ECL nanospheres was observed using a gel system imager and photographed (Fig. 2P). The ultrafiltrate containing free TCPP and DNA strands displayed red fluorescence, which disappeared after the uncoated TCPP particles were washed out. In contrast, the concentrate containing the DNA ECL nanosphere retained the red fluorescence from TCPP. Last, CLSM results confirmed the TCPP loading, as the DNA helical nanospheres showed no fluorescence, while the DNA ECL nanospheres displayed prominent red fluorescence (fig. S4). The four DNA ECL nanospheres used to detect *S. aureus*, MRSA, *P. aeruginosa*, and *K. pneumoniae*, were validated using agarose gel electrophoresis (fig. S5). These results collectively demonstrate the successful synthesis of the DNA ECL nanosphere, confirming its potential for subsequent applications.

ECL was used to monitor the TCPP release triggered by the binding of *S. aureus* to the DNA ECL nanosphere, providing preliminary validation of the feasibility of the assay (Fig. 2Q). After incubating *S. aureus* with the DNA ECL nanosphere for 40 min, the ECL signal increased significantly with an increase in the *S. aureus* concentration. These results suggest that the DNA ECL nanosphere can be used to homogeneously detect bacterium-aptamer interactions, offering a promising approach for developing aptamer-based assays for disease markers.

### Possible ECL mechanism of the NanoCe-S_2_O_8_^2−^-TCPP ECL system

**Fig. 3.**
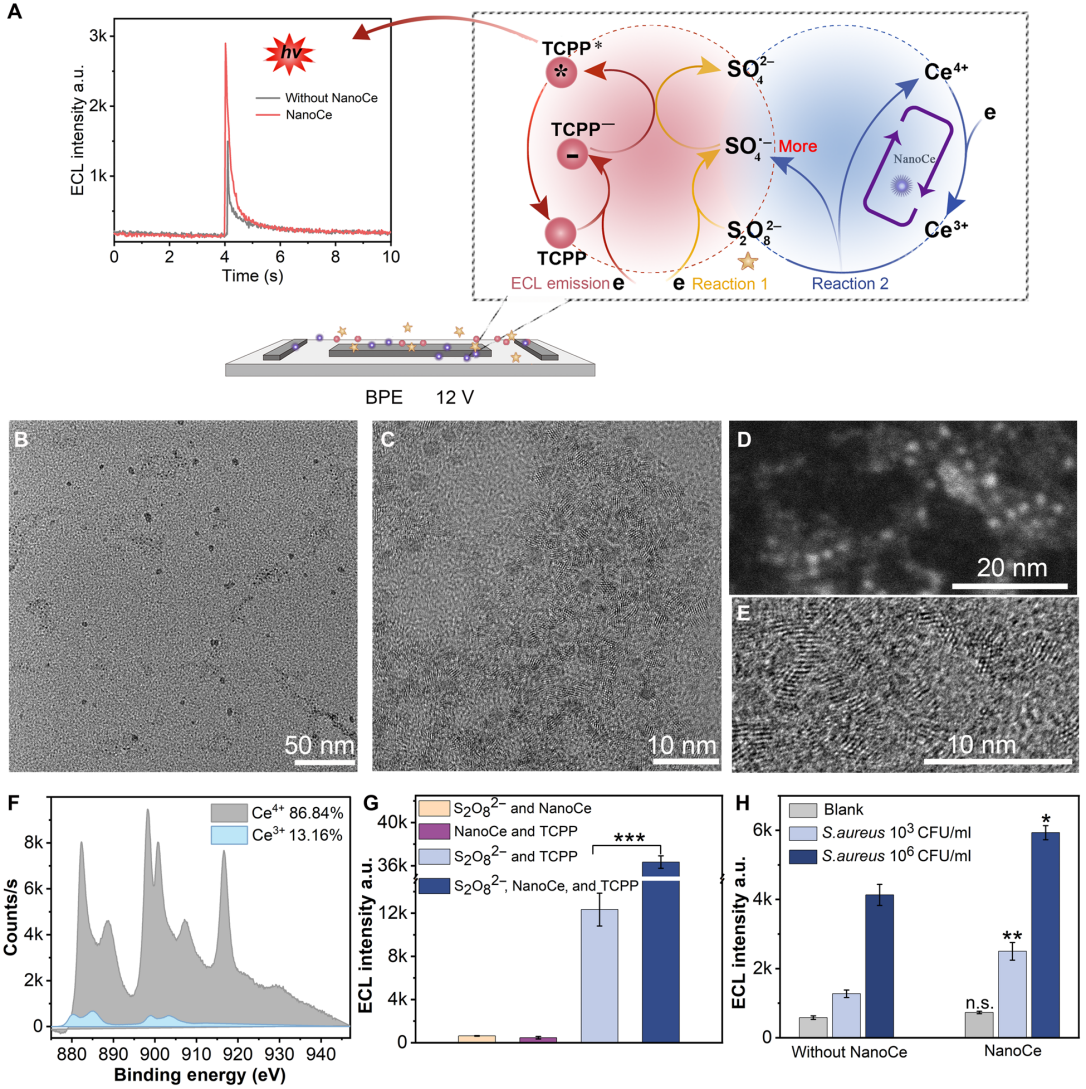
Characterization of NanoCe and ECL mechanism of NanoCe-S_2_O_8_^2−^-TCPP system. (**A**) Schematic illustration of the ECL mechanism of NanoCe-S_2_O_8_^2−^-TCPP system. BPE, bipolar electrode. (**B** and **C**) TEM images of NanoCe. (**D**) HAADF-TEM image of NanoCe. (**E)** TEM image of the lattice structure of NanoCe. (**F**) Nonlinear least squares fitting spectrum of Ce3d. (**G**) The ECL results of NanoCe, S_2_O_8_^2−^, and TCPP (1.2 μg/ml). (**H**) Validation of signal amplification in the NanoCe-S_2_O_8_^2−^-TCPP system through incubation with DNA ECL nanosphere and *S. aureus*. The experiments were repeated for three times (*n* = 3), and data were presented as means ± SD. Statistical analysis comparing without NanoCe versus NanoCe for the blank, 10^3^ CFU/ml, and 10^6^ CFU/ml groups. *P < 0.05; **P < 0.01; ***P < 0.001; n.s., not significant. a.u., arbitrary units.

Several studies have shown the ECL mechanisms of polyamide-amine dendritic polymer–enhanced TCPP (*34*), the ECL mechanism of S_2_O_8_^2−^-TCPP (*36*), and the ECL mechanism of Ce^3+^/Ce^4+^–coupled reduction of S_2_O_8_^2−^ (*32*). On the basis of these studies, the potential ECL mechanisms of the NanoCe-S_2_O_8_^2−^-TCPP system were investigated (Fig. 3A). In the absence of a coreactant, the ECL signals emitted from TCPP were hindered. However, when S_2_O_8_^2−^ was introduced as the coreactant, SO_4_^•−^ radicals were generated because of the reduction of S_2_O_8_^2−^ on the electrode surface. These radicals react with TCPP^−^ to produce the ECL signal. Furthermore, the incorporated NanoCe notably amplified the ECL signal as it can electrocatalytically reduce S_2_O_8_^2−^ to produce abundant SO_4_^•−^ radicals. Conversely, instead of amplifying the ECL signal of TCPP, free Ce^3+^ induced a quenching effect due to the coordination interaction between metal ions and TCPP (fig. S6A) (*37*). In summary, the synthesis and introduction of NanoCe are crucial, and the possible reaction mechanism can be proposed as follows: In reaction 1, S_2_O_8_^2−^ is reduced to SO_4_^•−^ on the electrode surface. In reaction 2, the Ce^3+^/Ce^4+^ redox couple facilitates the reduction of S_2_O_8_^2−^, leading to the production of additional SO_4_^•−^ radicals through a facile reaction between S_2_O_8_^2−^ and Ce^3+^. During the ECL emission process, TCPP is electrochemically reduced to TCPP^−^ on the electrode surface, and the TCPP^−^ then reacts with SO_4_^•−^ to form excited TCPP* molecules, resulting in light emission.

Reaction 1

$$S_{2}{O_{8}}^{2-}+e^{-}\to {{\text{SO}}_{4}}^{2-}+{{\text{SO}}_{4}}^{\cdot -}$$

Reaction 2

$${\text{Ce}}^{4+}+e^{-}\to {\text{Ce}}^{3+}$$

$${\text{Ce}}^{3+}+S_{2}{O_{8}}^{2-}\to {\text{Ce}}^{4+}+{{\text{SO}}_{4}}^{2-}+{{\text{SO}}_{4}}^{\cdot -}$$

ECL emission

$$\text{TCPP}+e^{-}\to {\text{TCPP}}^{-}$$

$${{\text{SO}}_{4}}^{\cdot -}+{\text{TCPP}}^{-}\to {\text{TCPP}}^{*}+{{\text{SO}}_{4}}^{2-}$$

$${\text{TCPP}}^{*}\to \mathrm{hv}+\text{TCPP}$$

### Characterization of NanoCe and verification of the signal amplification

Characterization of the morphology and dimensions of NanoCe using high-resolution TEM and HAADF revealed that NanoCe exists as uniform spheres with an average diameter of ~3 nm (Fig. 3, B to D). The lattice structure of NanoCe was clearly observed (Fig. 3E). The surface chemical composition and valence states of NanoCe were analyzed using x-ray photoelectron spectroscopy (XPS), which revealed the presence of Ce, C, S, Na, and O (fig. S6B) (*38*). To further investigate the mechanism of NanoCe as an accelerator of electrocatalysis, we examined the oxidation states of cerium. The Ce3d spectrum showed the coexistence of Ce^3+^ and Ce^4+^ on the surface of NanoCe (fig. S6C). Applying nonlinear least squares fitting to the Ce3d spectrum using the Avantage software, the ratio of Ce^3+^ (13.16%) to Ce^4+^ (86.84%) was determined (Fig. 3F). The coexistence of Ce^3+^ and Ce^4+^ increases the efficiency of the electrocatalytic reaction in the initial stage. These results confirm that NanoCe was successfully synthesized and can potentially act as an effective coreaction accelerator (*39*).

We also investigated whether NanoCe facilitates the amplification of the TCPP-S_2_O_8_^2−^ ECL system. The ECL signal was nearly undetectable in the absence of TCPP or S_2_O_8_^2−^. The ECL signal was increased noticeably in the NanoCe-incorporated TCPP-S_2_O_8_^2−^ system compared to the TCPP-S_2_O_8_^2−^ system only (Fig. 3G). In the complete *S. aureus* detection system, the addition of NanoCe did not significantly increase the background signal but enhanced the *S. aureus* detection signal (Fig. 3H). These results suggest that NanoCe can accelerate the TCPP-S_2_O_8_^2−^ ECL system, enhancing the ECL signal and thereby improving the sensitivity and detection range of the assay.

### Application validation of the ECL microfluidic arrays

**Fig. 4.**
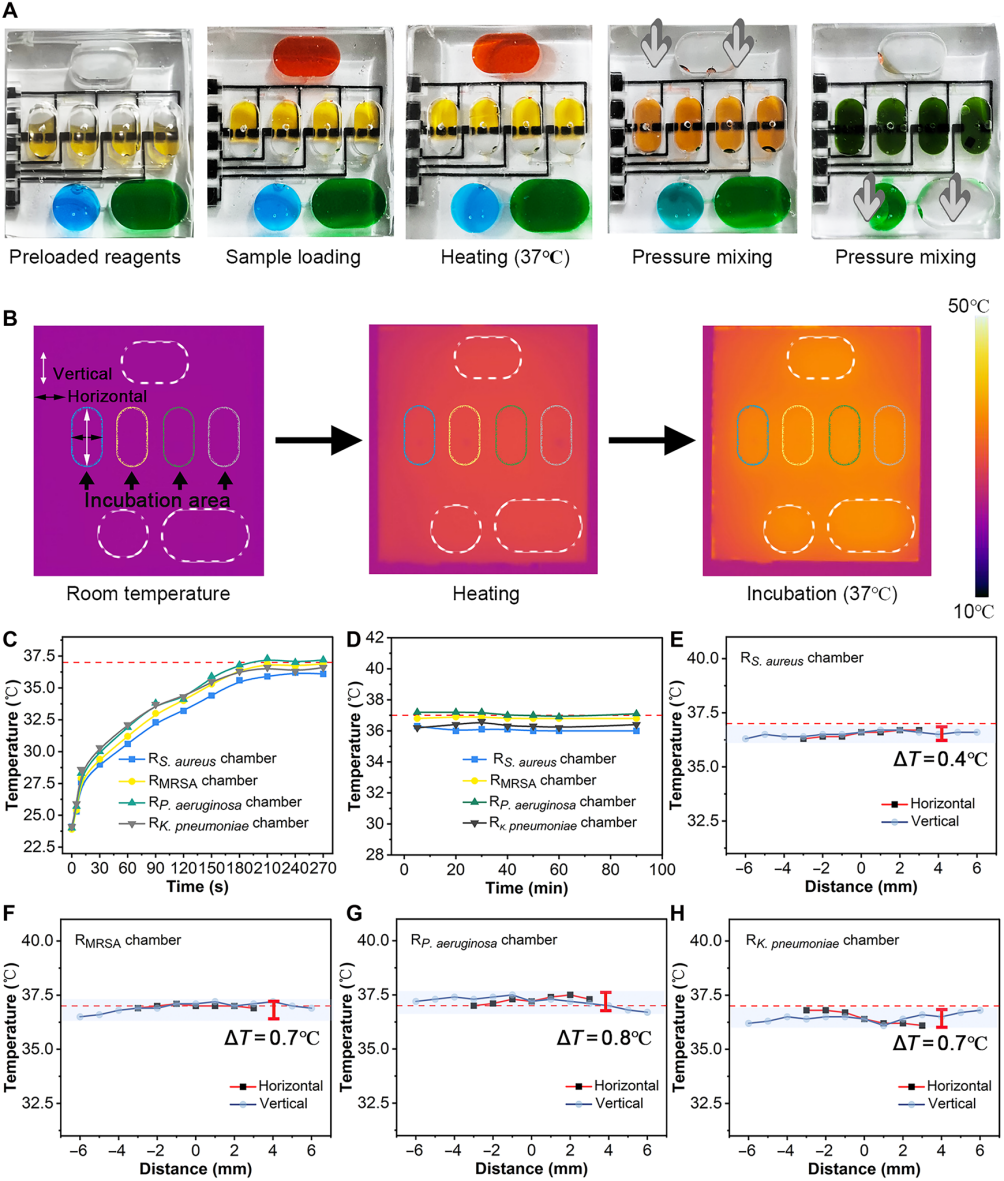
Design and application validation of the ECL microfluidic arrays. (**A**) Liquid stability testing in different steps. (**B**) Heated area and thermal imaging of the microfluidic chip. (**C**) Temperature variation in the R*_S. aureus_* chamber, R_MRSA_ chamber, R*_P. aeruginosa_* chamber, and R*_K. pneumoniae_* chamber during heating. (**D**) Temperature change during 90 min of heating at 37°C. (**E** to **H**) Temperature variation at different sites within the R*_S. aureus_* chamber, R_MRSA_ chamber, R*_P. aeruginosa_* chamber, and R*_K. pneumoniae_* chamber during heating. R, reagent.

The microfluidic delivery of the samples and reagents and the temperature control in the incubation area are critical parameters. As shown in Fig. 4A, red, yellow, green, and blue solutions were injected into the sample, reagents, coreactant, and accelerator chambers, respectively, to verify that the samples, coreactant, and accelerator could be effectively mixed with the diagnostic reagents in the reagent chambers under the stepper motor pressure. After incubation at 37°C for 1 hour, the liquid remained stable without reflux. Subsequently, applying pressure with a stepper motor facilitated the effective mixing of solutions within the chambers. As shown in fig. S7, when the stepper motor’s downward pressure speed was 0.1 mm/s or less, liquids from the sample and coreaction/accelerator chambers entered the reagent chambers effectively, resulting in uniform mixing. Furthermore, a speed of 0.1 mm/s produced the shortest processing time. In contrast, uneven liquid distribution was observed at a speed of 0.15 mm/s. Thus, this study identified a speed of 0.1 mm/s and a duration of 30 s as the optimal conditions for downward pressure. These results prove that sample detection can be potentially automated in the microfluidic chip.

To further validate the feasibility of the chip, a thermal imaging camera was used to monitor its temperature. The thermal imaging of the microfluidic chip confirmed its temperature distribution, which fluctuated around 37°C (Fig. 4B). After 3 min of heating, the temperature of the four reagent chambers stabilized around 37°C (Fig. 4C), without any notable fluctuations throughout the 90-min heating process at 37°C (Fig. 4D). The temperature difference between the internal positions of the four reagent zones ranged from 0.4° to 0.8°C (Fig. 4, E to H), indicating favorable temperature stability. To evaluate the effect of temperature fluctuations on detection performance, we tested ECL signals at low and high bacterial concentrations across three temperatures (36.5°, 37°, and 37.5°C). The results showed no statistical difference in ECL signals at any concentration (fig. S8), suggesting that the effect of temperature fluctuations within this range on the detection performance is minimal. These results demonstrate that the ECL microfluidic arrays can detect four targets simultaneously, thereby improving detection efficiency.

### Validation of aptamer for the specific recognition of bacterial pathogens

A key feature of this detection sensor is the specificity of the aptamer toward the target bacteria. To assess the feasibility of this strategy, this specificity was analyzed using fluorescence spectroscopy, flow cytometry, and CLSM. For this, specific aptamers were incubated with bacteria from clinical samples to identify the most promising candidates. The 6-carboxyfluorescein (6-FAM)–labeled aptamers (table S2) were incubated with the corresponding target bacteria [optical density at 600 nm (OD_600_) of 0.5], including *S. aureus*, MRSA, *P. aeruginosa*, and *K. pneumoniae*, at 37°C for 1 hour. A nonspecific bacterial mixture was used as a control (table S3).

**Fig. 5.**
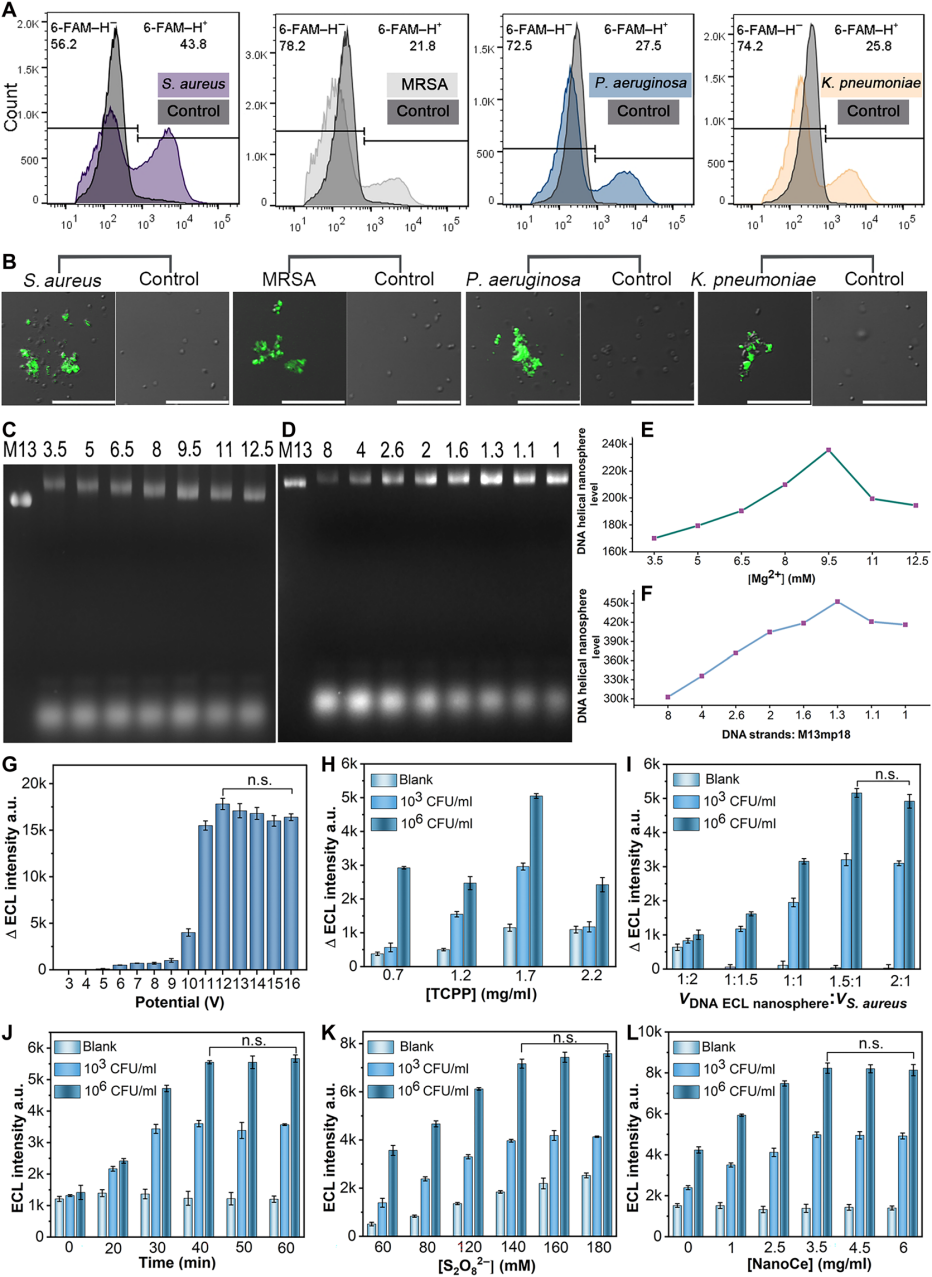
Results of aptamer binding to bacteria and optimization of experimental conditions. (**A**) Flow cytometry results of aptamer and bacteria binding using the Brilliant Green 488 dye channel. (**B**) Fluorescence images of bacteria and aptamer binding (partial enlargement). (**C**) Electropherogram at different concentrations of Mg^2+^ (millimolar). (**D**) Electropherogram at different ratios of DNA strands and M13mp18. (**E** and **F**) Digital quantification of the electropherograms using ImageJ. (**G**) ECL detection results at different detection voltages. (**H**) ECL detection results at different concentrations of TCPP. (**I**) ECL detection results at different ratios of DNA ECL nanosphere and *S. aureus*. (**J**) ECL detection results at different incubation times. (**K**) ECL detection results at different concentrations of S_2_O_8_^2−^. (**L**) ECL detection results at different concentrations of NanoCe. ∆ECL = ECL_40 min_ − ECL_0 min_. Scale bars, 20 μm. The experiments were repeated for three times (*n* = 3) and data were presented as means ± SD. n.s., not significant. a.u., arbitrary units.

Initial screening of the aptamer was performed using fluorescence spectroscopy (fig. S9). While the fluorescence spectra of the nonspecific bacterial mixture exhibited almost no responses, the fluorescence intensities were notably increased when *S. aureus*, MRSA, *P. aeruginosa*, and *K. pneumoniae* were added to the specific aptamer reaction system. In particular, *S. aureus* aptamer 1, MRSA aptamer 2, *P. aeruginosa* aptamer 2, and *K. pneumoniae* aptamer 1 exhibited increased fluorescence. These four highly fluorescent aptamers were subsequently analyzed by flow cytometry and CLSM. Incubation of the aptamer with their respective target bacteria resulted in an increase in fluorescence intensity, indicating a strong binding affinity compared with the control group (Fig. 5A). Specifically, the right-shifted peak observed in the sample suggests that the 6-FAM–labeled aptamers could successfully bind to the bacteria, resulting in a change in fluorescence intensity. No change in fluorescence intensity was observed in the control group, further confirming the specific binding between the aptamers and their target bacteria. In the dark field, the green, fluorescent dots from 6-FAM overlapped with specific bacteria in the bright field, unlike that in the control group (Fig. 5B and fig. S10). These results demonstrated that *S. aureus* aptamer 1, MRSA aptamer 2, *P. aeruginosa* aptamer 2, and *K. pneumoniae* aptamer 1 exhibited excellent target specificity and, hence, were selected for the subsequent experiments.

### Optimization of the experimental conditions

To achieve optimal detection performance of the ECL microfluidic arrays based on DNA ECL nanosphere, several experimental conditions were systematically investigated, including the ECL detection voltage; the concentrations of Mg^2+^, TCPP, S_2_O_8_^2−^, and NanoCe; the concentration ratio of DNA strands to M13mp18; the volume ratio of DNA ECL nanosphere to *S. aureus*; and the incubation time (Fig. 5, C to L). We selected the experimental conditions at which the ECL intensity reached a plateau or its maximum value. The DNA helical nanosphere was optimized at a Mg^2+^ concentration of 9.5 mM and DNA strands to M13mp18 ratio of 1.3 (Fig. 5, C to F). Additionally, the voltage between the working and counter electrodes was set at 12 V as the highest ECL signal was obtained at this voltage (Fig. 5G). For the remaining conditions, the strongest signal intensity was observed at a TCPP concentration of 1.7 mg/ml (Fig. 5H), a volume ratio of DNA ECL nanosphere to *S. aureus* of 1.5:1 (Fig. 5I), and an incubation time of 40 min (Fig. 5J). The optimal concentrations of S_2_O_8_^2−^ and NanoCe were 140 mM and 3.5 mg/ml, respectively (Fig. 5, K and L). Moreover, the greatest variation in the ECL signal intensity was observed across different bacterial concentrations. On the basis of these results, the optimized experimental conditions mentioned above were applied to the multitarget homogeneous ECL sensor.

### The performance of the ECL microfluidic arrays for multibacterial analysis

**Fig. 6.**
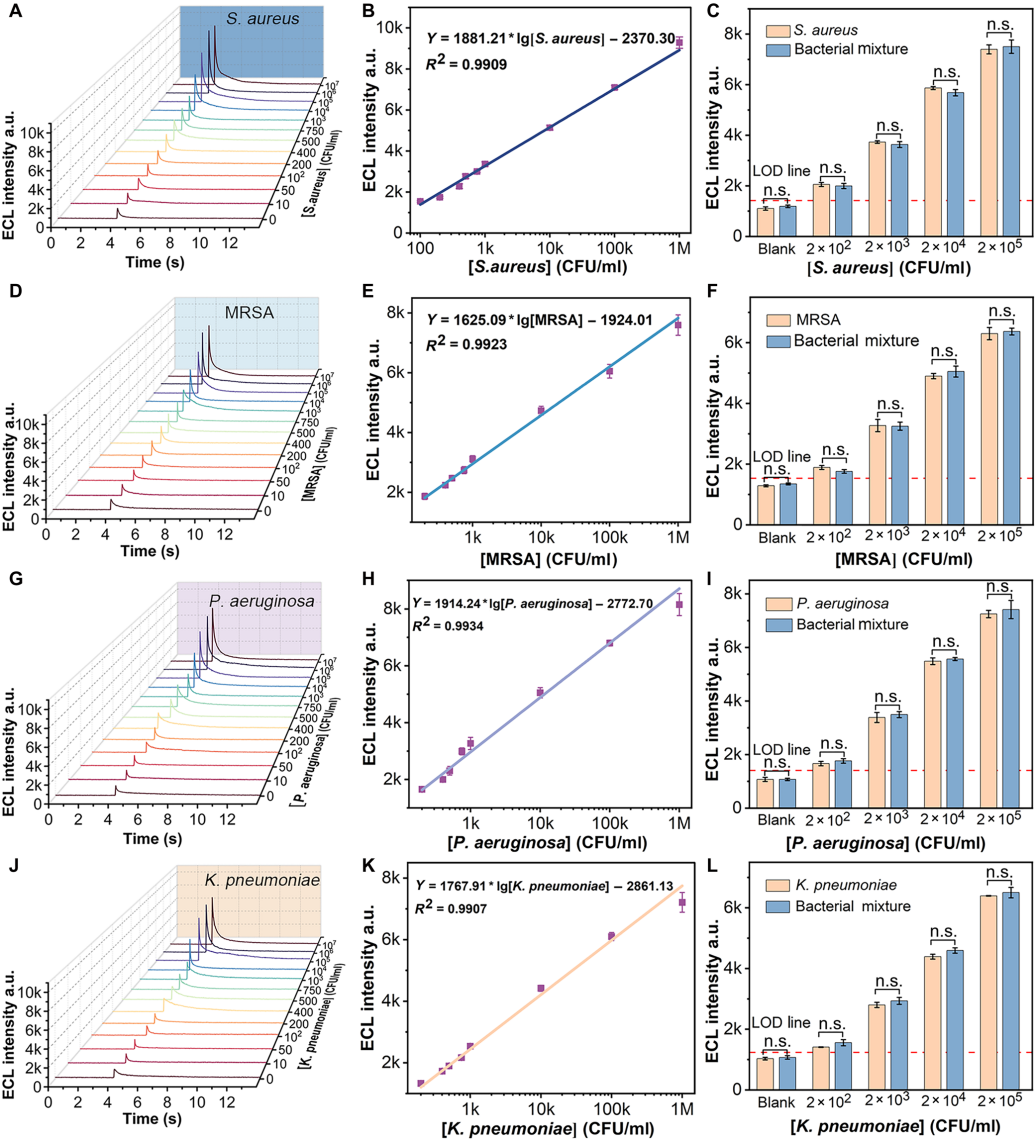
Performance of the ECL microfluidic arrays for different types of bacterial analyses. (**A**, **D**, **G**, and **J**) ECL responses of the microfluidic arrays toward different concentrations of *S. aureus*, MRSA, *P. aeruginosa* and *K. pneumoniae*. (**B**, **E**, **H**, and **K**) Linear analysis of the ECL detection results. (**C**, **F**, **I**, and **L**) Selectivity of the microfluidic arrays. Bacterial mixture: Different concentrations of bacteria were mixed (table S6), with the concentration of nontarget bacteria being 10 times higher than the target bacteria, to construct an artificial complex sample. The experiments were repeated for three times (*n* = 3) and data were presented as means ± SD. n.s., not significant. a.u., arbitrary units.

The next objective was to evaluate the detection sensitivity and linear range of the multibacterial ECL arrays using *S. aureus*, MRSA, *P. aeruginosa*, and *K. pneumoniae* as bacterial pathogens. A range of bacterial concentrations was detected, and the corresponding ECL intensity was measured by introducing S_2_O_8_^2−^ and NanoCe into the reaction solution. As expected, the ECL intensity increased with the increasing concentrations of the target bacteria (Fig. 6, A, D, G, and J; and fig. S11). Figure 6 (B, E, H, and K) shows a linear correlation between the ECL intensity and the logarithmic values of the target concentrations. The linear regression equations for each pathogen were as follows: *Y* = 1881.21 * lg[*S. aureus*] − 2370.3 [coefficient of determination (*R*^2^) = 0.9909]; *Y* = 1625.09 * lg[MRSA] − 1924.01 (*R*^2^ = 0.9923); *Y* = 1914.24 * lg[*P. aeruginosa*] − 2772.70 (*R*^2^ = 0.9934); and *Y* = 1767.91 * lg[*K. pneumoniae*] − 2861.13 (*R*^2^ = 0.9907). The LOD values for the four pathogens were estimated as follows: 50 CFU/ml for *S. aureus*, 100 CFU/ml for MRSA, 80 CFU/ml for *P. aeruginosa*, and 95 CFU/ml for *K. pneumoniae* (based on the ECL intensity of the blank samples plus three times of the SD). The LOQ values for the four pathogens were estimated as follows: 100 CFU/ml for *S. aureus*, 200 CFU/ml for MRSA, 200 CFU/ml for *P. aeruginosa*, and 200 CFU/ml for *K. pneumoniae.* To further highlight the merits of this detection platform, its performance parameters, such as LOD, linear range, detection time, and washing steps, were compared with those of other bacterial detection methods and S_2_O_8_^2−^-TCPP ECL sensor (table S4 and fig. S12). This method offers key advantages, including a low LOD, a wide log-linear range, no washing step, simple operation, and a short analysis time. Furthermore, compared to traditional detection methods, this study presents additional advantages, including reduced analysis time and simplified operation, thereby offering promising prospects for clinical applications (table S5).

To evaluate the specificity and feasibility of this method, a series of tests were performed using blank controls, target bacteria, and mixtures of target and nontarget bacteria (table S6). The hybridization solution and bacterial mixtures without the target bacteria (blank group) exhibited minimal ECL signal intensity with no statistical change (Fig. 6, C, F, I, and L). In contrast, samples containing the target bacteria exhibited stronger ECL signals, which increased progressively with higher bacterial concentrations. No statistical changes in ECL signal intensity were observed between samples with the same bacterial concentration and their respective bacterial mixtures. The ECL signal intensities for both target bacteria and bacterial mixtures remained within the 95% prediction interval of the regression curve (fig. S13). These results demonstrate that the ECL platform exhibits excellent specificity, enabling the accurate identification of target bacteria even in mixed bacterial solutions.

We validated the reproducibility of DNA ECL nanosphere within the same batch and across different batches (fig. S14, A and B). The results showed that different bacterial concentrations could be accurately identified using reagents from either the same or different batches. Despite differences in ECL signals at the same bacterial concentration (between different batches), accurate calculations could be made using three-point calibration or an updated standard curve, consistent with clinical practice. Additionally, the DNA ECL nanosphere exhibited excellent detection stability after being stored at −20°C for 28 days (fig. S14C).

### Clinical application of the ECL microfluidic arrays

The reliability and feasibility of the developed method were analyzed using 27 clinical samples, including 9 samples each from urine, blood, and pleuroperitoneal effusion. These samples were analyzed using the DNA ECL nanosphere–based microfluidic assays, and the results were compared with those obtained using digital polymerase chain reaction (PCR; figs. S15 to S20).

**Fig. 7.**
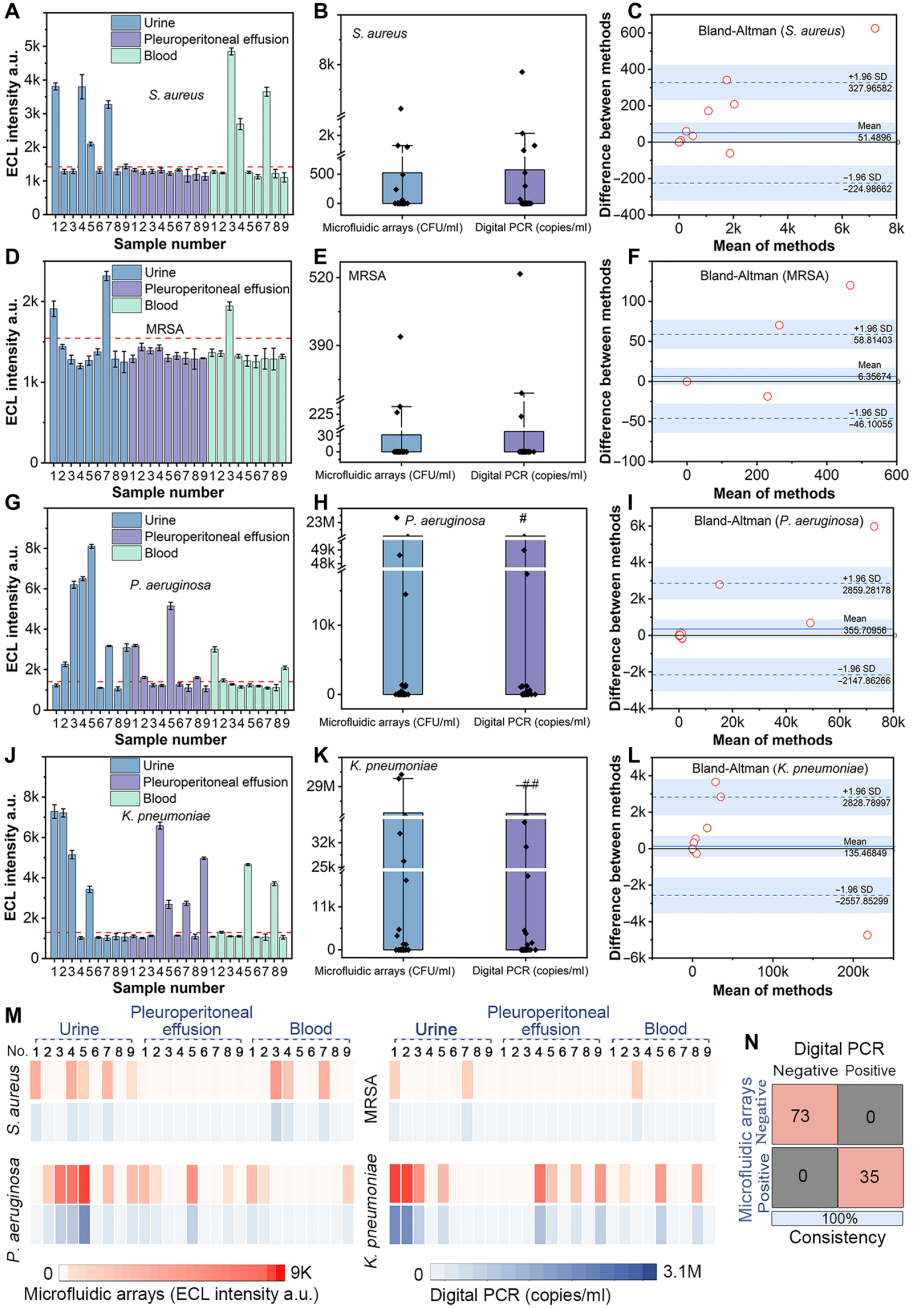
Results of clinical sample analysis. (**A**, **D**, **G**, and **J**) Detection of *S. aureus*, MRSA, *P. aeruginosa*, and *K. pneumoniae*, respectively, in clinical samples using the microfluidic arrays, showing ECL signal intensity. (**B**, **E**, **H**, and **K**) Comparison of bacterial concentrations obtained with microfluidic arrays and digital PCR. The data points exceeding the detection limit (denoted as “#”) were truncated at the detection limit. (**C**, **F**, **I**, and **L**) Bland-Altman analysis comparing bacterial concentrations from the microfluidic arrays and digital PCR. Removing data points above the upper detection limit. (**M**) Comparison of results from the microfluidic arrays and digital PCR using a heatmap. The data points exceeding the detection limit were truncated at the detection limit. (**N**) Evaluation of the sensitivity and specificity of the microfluidic arrays compared to digital PCR using a confusion matrix. The experiments were repeated for three times (*n* = 3), and data were presented as means ± SD. a.u., arbitrary units.

Figure 7 shows the detection results for *S. aureus*, MRSA, *P. aeruginosa*, and *K. pneumoniae* in the clinical samples using the ECL sensor (Fig. 7, A, D, G, and J). The ECL signal was clearly distinguishable in all bacteria-positive samples, while the bacteria-negative samples exhibited only faint ECL signals. The data distribution of the ECL sensor and digital PCR method in detecting different bacterial concentrations was visualized using boxplots with overlapping dots (Fig. 7, B, E, H, and K). The relationship between the two methods was further assessed using Spearman’s correlation coefficient and regression analysis. The Spearman correlation coefficients were 0.9991, 0.9980, 0.9976, and 1 for *S. aureus*, MRSA, *P. aeruginosa*, and *K. pneumoniae*, respectively, and the *R*^2^ values of regression analysis were 0.9983, 0.9829, 0.9984, and 0.9994 (fig. S21), respectively, indicating a very high degree of agreement between the ECL assay and digital PCR. Additionally, the consistency between the two assays was evaluated using the Bland-Altman analysis (Fig. 7, C, F, I, and L). The results showed that 96.3% (*S. aureus*), 92.59% (MRSA), 96.15% (*P. aeruginosa*), and 92% (*K. pneumoniae*) of the data points fell within the means ± 1.96 SDs of the difference, suggesting good agreement between the methods, with the observed differences falling within acceptable limits. The heatmap results showed that the ECL signal intensity followed the same trend as the bacterial concentration for digital PCR (Fig. 7M). A qualitative comparison between the ECL assay and the digital PCR results was conducted using a confusion matrix (Fig. 7N). The ECL method accurately identified both positive and negative bacterial samples from urine, pleuroperitoneal effusions, and blood, showing 100% agreement with the digital PCR results. Overall, these findings confirm that the ECL method is highly reliable for detecting *S. aureus*, MRSA, *P. aeruginosa*, and *K. pneumoniae*, with strong concordance to digital PCR. The homogeneous ECL microfluidic arrays require <1 hour, with simple operation and low cost, highlighting their potential for practical applications.

## DISCUSSION

In this study, a homogeneous ECL microfluidic array was developed on the basis of DNA ECL nanospheres, NanoCe, and microfluidic technology for the rapid and synchronous detection of multiple bacterial pathogens, including *S. aureus*, MRSA, *K. pneumoniae*, and *P. aeruginosa*. The method used the NanoCe-S_2_O_8_^2−^-TCPP ECL system, enabling the sensitive and specific detection of bacterial pathogens in various biological samples, including urine, pleuroperitoneal effusion, and blood. The assay time is ~45 min, which expedites results and substantially reduces the sample turnaround time.

In terms of detection time, traditional methods such as automated bacterial identification instruments and plate counting required over 18 hours (*40*). Conversely, digital PCR (*41*) is highly sensitive and can provide results within just 3 to 5 hours, but it has high requirements and costs. Similarly, MALDI-TOF MS, another effective tool for rapid bacterial identification (*13*), requires costly instrumentation and substantial bacterial samples. Several bacterial sensing platforms have been developed to improve the speed and accuracy of identification (*19*, *40*, *42*, *43*), such as electrochemiluminescent sensors, electrochemical sensors, colorimetric sensors, and fluorescent sensors. However, these methods are often limited by long detection times, restricted sensitivity, single-pathogen detection, or the need for amplification processes. Furthermore, accurate measurement of bacterial concentration not only aids physicians in effectively identifying pathogens and differentiating them from potential contaminating bacteria but also assists in evaluating the severity of infections. This importance further emphasizes the limitations of qualitative detection methods. In contrast, this ECL microfluidic array integrates rapid detection, high sensitivity, and user-friendliness, providing an effective strategy for the quantitative detection of multiple bacteria.

A key feature of the homogeneous ECL sensor is the structure of the DNA ECL nanosphere and their unique “close-open” property. First, the diameter and uniformity of the nanosphere affect their contact area with target molecules, the loading capacity of TCPP, and the stability of reagents, which ultimately affect the sensor’s detection performance. In this study, the uniform size distribution of the synthesized DNA ECL nanosphere ensured stable loading of TCPP, achieving fast response and high accuracy in bacterial detection. Second, the close-open property is activated through a conformational change that occurs when the aptamers on the nanosphere bind to the bacteria. This process releases the TCPP within the nanosphere and triggers enhanced ECL. This ensures the targeted release of ECL emitters and minimizes background interference, a common challenge in multitarget detection platforms. Furthermore, the “off-on” mode does not require a wash step, unlike traditional ECL assays, which expedites detection. Last, the use of aptamers for target recognition reduces costs by eliminating the need for expensive antibodies. To further improve detection sensitivity and broaden the range of bacterial recognition, future aptamer screening should focus on expanding the types of target bacteria and selecting aptamers with stronger affinity.

The design leverages the Ce^4+^/Ce^3+^ redox pair within the NanoCe, which effectively reduces S_2_O_8_^2−^, leading to the generation of a higher number of SO_4_^•−^ radicals. This process substantially amplifies the ECL signal and enhances the assay sensitivity, owing to the excellent electrical conductivity and strong catalytic properties of NanoCe (*44*). The ratio of Ce^4+^/Ce^3+^ in NanoCe may play a critical role in modulating its catalytic efficiency (*45*, *46*). Therefore, further experiments should be conducted to investigate how variations in this ratio affect the S_2_O_8_^2−^-TCPP ECL signal to understand its impact on assay performance.

Last, we integrated the microfluidic chip into the ECL sensor to automate reagent, sample mixing, and incubation, simplifying the process. This operational simplicity reduces the time required to detect four bacteria simultaneously to just 45 min. Furthermore, slight temperature fluctuations in the microfluidic device do not affect the performance of the detection, a feature that allows the ECL sensor to be used outside traditional laboratory settings, such as on-site diagnostics or in areas with limited healthcare infrastructure.

The compositions of clinical samples, such as urine, blood, and thoracoabdominal effusions, differ in terms of their cell content, inorganic salt concentrations, metabolic products, protein types and concentrations, and inflammatory factors (*47*–*49*). In this study, as the samples are pretreated using methods, such as centrifugation, the ECL signal derived from the fluorescent dye TCPP remained unaffected by these variations. Additionally, as different sample types reflect various infection sites and pathological states, selecting the appropriate sample for testing is crucial for ensuring an accurate diagnosis. Although this study demonstrates high accuracy, further validation with a larger sample size is necessary to confirm the reliability and effectiveness of this method.

In conclusion, the ECL microfluidic arrays provide a rapid, reliable, and easy-to-use tool for detecting bacterial pathogens that could greatly enhance infection management and treatment outcomes. However, several challenges remain. While the system demonstrates high specificity and sensitivity, the ECL system needs further investigation to ensure consistent performance under various conditions. Furthermore, future research should also focus on expanding the range of detectable pathogens and further validating this platform in clinical settings.

## MATERIALS AND METHODS

### Materials and reagents

M13mp18 was acquired from Bayou Biolabs (USA). Cerium(III) nitrate hexahydrate [Ce(NO_3_)_3_·6H_2_O] and TCPP were obtained from Aladdin (Shanghai, China). Sodium bis(2-ethylhexyl) sulfosuccinate (C_20_H_37_NaO_7_S, AOT) was supplied by Sigma-Aldrich (USA). MgCl_2_·6H_2_O and DNA marker were procured from Sangon Biotechnology Co. Ltd. (Shanghai, China). The staple DNA strands provided in table S1 were synthesized and purified by Sangon Biotechnology Co. Ltd. (Shanghai, China). NaCl was purchased from Chengdu JinShan Chemical Co. Ltd. (Chengdu, China). The dialysis bag was purchased from MYM Biological Technology Company Limited (USA). The clinical samples were provided by the Laboratory of Molecular Diagnostics in Zhongshan City People’s Hospital (Zhongshan, China). This study was approved by the Biomedical Ethics Committee of Dermatology Hospital, Southern Medical University (approval number KY-2025-003). Bacteria and DNA ECL nanospheres were incubated in the hybridization buffer, including 20 mM tris-HCl, 0.1 M NaCl, and 5 mM MgCl_2_ (pH 7.4). The aluminum mold was fabricated by the Shenzhen Kotaixin Precision Co. (ShenZhen, China). All reagents were of analytical reagent grade and used directly without further purification. Milli-Q water (>18.2 megohms) was obtained from a Millipore Mill-Q purification system.

### Apparatus and characterization

The DNA helical nanosphere and DNA ECL nanosphere were synthesized by DLAB TC1000-G gene amplifier (Shanghai, China). TEM was conducted by FEI Talos 200S (USA). AFM images were obtained using the Bruker Dimension icon microscope (Germany). Confocal images were obtained using the Sunny Optical CLSM600 laser confocal microscopy (China). The UV-vis spectra were measured using the Lambda 365 UV/VIS spectrophotometer (PerkinElmer, USA). The fluorescence spectra and fluorescence values were obtained using an RF-6000 fluorescence spectrophotometer (Shimadzu, Japan). The ζ-potential was determined using a Litesizer 500 (Anton Paar, Austria). XPS (Thermo Scientific K-Alpha+, USA) was used to monitor the elemental composition of NanoCe. The conductive carbon ink for the electrodes was obtained from Xuzhou Bohui New Materials Technology Co. Ltd. (Xuzhou, China). The digital PCR results were obtained using the Droplet Digital PCR system (D3200, Pioneering Gene Technology, Hangzhou, China).

### Preparation of the DNA helical nanosphere and DNA ECL nanosphere

Within the DNA helical nanospheres, the polyT sequences at the 5′ and 3′ ends of the aptamer undergo complementary base pairing with the polyA sequences of the fixed short DNA strands, referred to as a-lock and b-lock. The aptamer sequences, highlighted in red and blue, respectively, form complementary base pairs with their corresponding variable short DNA strands of the same color. This design ensures that the aptamer reliably binds to both a-lock and b-lock chains, thereby establishing a stable chain structure through complementary base pairing. By replacing the aptamer and five variable short DNA strands, diagnostic reagents capable of specifically recognizing *S. aureus*, MRSA, *P. aeruginosa*, and *K. pneumoniae* can be prepared. The relevant DNA sequences are detailed in table S1.

To prepare the DNA helical nanosphere, 172 DNA strands and M13mp18 were annealed at a 1.3:1 molar ratio in TAE/Mg^2+^ buffer (40 mM tris-HCl, 20 mM acetic acid, 2 mM EDTA, and 9.5 mM magnesium acetate; pH 8) using a gene amplifier. The cycling protocol involved an initial temperature ramp to 95°C, held for 2 min, followed by a gradual cooling from 68° to 4°C at a rate of 2°C/min. The resulting DNA helical nanospheres were purified using Amicon Ultra 2-ml 100-K centrifugal filters to remove the excess DNA strands. The synthesis of DNA ECL nanosphere is similar to that of DNA helical nanosphere. Both use the same DNA concentration, buffer, and preparation steps. TCPP (1.7 mg/ml) was added as the final reagent, mixed thoroughly, and annealed gradually to achieve ECL properties.

### Synthesis of NanoCe

NanoCe was synthesized using the microemulsion method (*39*), consisting of surfactant AOT (1.5 g), toluene, and water. After dissolving AOT in 50 ml of toluene, 2.5 ml of 0.1 M aqueous Ce(NO_3_)_3_·6H_2_O solution was added, and the mixture was stirred for 45 min. Then, 5 ml of 30% hydrogen peroxide (H_2_O_2_) solution was added dropwise, and the reaction was incubated for 1 hour. The reaction mixture was allowed to separate into two layers. The upper layer consisted of toluene with nonagglomerated NanoCe, while the lower layer was the aqueous phase. Last, NanoCe particles were precipitated by adding 30% ammonia solution, followed by multiple washes with acetone and water to remove the surfactant completely. The obtained particles were then resuspended in deionized water to the desired concentration.

### Fluorescence-based validation of the binding of the aptamer to the bacteria

The pathogens used in the experiments were sourced from a clinical laboratory. Eight aptamers labeled with the 6-FAM marker were introduced, including two aptamers each for *S. aureus* (*50*, *51*), MRSA (*52*–*54*), *P. aeruginosa* (*55*–*58*), and *K. pneumoniae* (*59*, *60*) (see table S2 for details).

The bacterium-aptamer binding was verified using the spiking method (table S3). A nontarget bacterial mixture was used as the negative control. In summary, 1 μM 6-FAM–labeled aptamer was incubated with the bacteria (OD_600_ of 0.5) in 200 μl of hybridization buffer for 1 hour at 37°C. After incubation, the mixture was centrifuged to remove the unbound aptamer from the supernatant. The bacterial solution was washed thrice and resuspended in phosphate-buffered saline (PBS). The fluorescence intensity of the resuspended samples was measured using a fluorescence spectrophotometer at an excitation wavelength of 494 nm.

Subsequently, CLSM was used to analyze the four high-fluorescence aptamers, including *S. aureus* aptamer 1, MRSA aptamer 2, *P. aeruginosa* aptamer 2, and *K. pneumoniae* aptamer 1. For these analyses, 1 μM 6-FAM–labeled aptamer was incubated with the bacterial suspension (OD_600_ of 0.5) at 37°C for 1 hour. After incubation, the mixture was centrifuged, washed thrice, and resuspended in PBS. Bacterial imaging was performed using a confocal microscope (Sunny Optical CLSM600) at an excitation wavelength of 488 nm and visible light. The fluorescence intensity of the bacteria was monitored using a FACSCalibur cytometer (BD Biosciences, USA). The results were analyzed using the FlowJo software.

### Preparation of the microfluidic chip

The microfluidic detection chip was constructed with a silicon substrate, a carbon electrode, and a PDMS layer containing a reaction chamber. In the first step, the silicon substrate (35 mm length by 30 mm width) was completely immersed in a 1 M HCl solution and subjected to ultrasonic cleaning for 1 hour. Then, the substrate was washed with ultrapure water, followed by NaOH solution (1 M), and lastly washed again with ultrapure water. The silicon substrate must be maintained in a pristine and dust-free condition. The driving and working electrodes (carbon ink) were screen printed on the surface of the silicon substrate using a custom screen that was dried in a convection oven at 70°C before use. The silicon substrate used for printing the electrodes was stored for later use.

Next, the PDMS chip was designed and fabricated. The structure of the chip mold for the PDMS layer was designed using the SolidWorks software package. The diameter and depth dimensions of the sample, reagent, coreactant, and accelerator chambers were 11 and 3 mm, 12.5 and 3 mm, 9 and 3 mm, and 8 and 1 mm, respectively. PDMS was combined with the hardener at a ratio of 10:1. Subsequently, the mixture was poured into an aluminum mold and degassed once more. Subsequently, the mixture was subjected to a 20-min curing process at 120°C in a convection oven, after which it was removed from the mold. The cured PDMS layer was punched to create a circular aperture with a diameter of 1 mm. Subsequently, the PDMS layer and silicon substrate were treated with oxygen plasma for 60 s using a PDC-MG plasma cleaner, after which they were bonded together for 12 hours. Last, the detection reagent of the DNA ECL nanosphere, coreactant, and accelerator were injected into the reagent, coreactant, and accelerator chambers, respectively.

### Protocols for the ECL microfluidic detection

According to the homogeneous reaction strategy without washing, six reagents were preloaded into the following chambers of the fabricated microfluidic chip: Reagent*_S. aureus_*, Reagent_MRSA_, Reagent*_P. aeruginosa_*, Reagent*_K. pneumoniae_*, coreactant, and accelerator. The reagents were as follows: 90 μl of *S. aureus*/DNA ECL nanosphere (R*_S. aureus_*), MRSA/DNA ECL nanosphere (R_MRSA_), *P. aeruginosa*/DNA ECL nanosphere (R*_P. aeruginosa_*), and *K. pneumoniae*/DNA ECL nanosphere (R*_K. pneumonia_*); 40 μl of NanoCe; and 180 μl of K_2_S_2_O_8_. The workflow of the detection system is illustrated in Fig. 1. Initially, the sample was injected into the designated sample chamber. Subsequently, a stepper motor was used to meticulously inject 240 μl of the sample into the four reagent chambers, using a slow and controlled pressure. The microfluidic chip was heated to 37°C and maintained at this temperature for 40 min to ensure stable reaction progress. Following incubation, K_2_S_2_O_8_ and NanoCe were added to the four reagent chambers via the stepper motor, and slow downward pressure was applied multiple times to ensure thorough mixing of the reagents. Last, the microfluidic chip was inserted into the ECL detection auxiliary device, and the ECL reaction was driven via a 12-V dc voltage. The operating video of the device is presented in movie S1.

### Sample collection

In this study, all clinical samples, including blood, urine, and pleuroperitoneal effusion, were collected and processed in strict accordance with the standardized procedures. Blood samples were drawn from patients’ veins using aseptic techniques and then centrifuged at 3000 rpm for 5 min to separate the plasma from the blood cells. Urine samples were tested directly. However, if they contained visible red blood cells (RBCs) or clots, then they were first centrifuged (3000 rpm, 5 min) before testing. Pleural and peritoneal effusion samples were collected via sterile puncture. If the sample contains RBCs or clots, then it must also be centrifuged before testing. When the bacterial concentration exceeds the maximum detection range, it is advisable to dilute the sample before retesting. All samples should either be tested immediately or stored at −20°C to ensure stability.

### Statistical analysis

The statistical differences between the two groups were assessed using two-sided *t* test. Multiple comparisons were made using one-way analysis of variance (ANOVA) with post hoc correction. The consistency between the ECL system and digital PCR was assessed using correlation analysis, regression analysis, and Bland-Altman analysis. Pearson’s correlation was used for normally distributed data, while Spearman’s rank correlation was used for nonnormally distributed data. The statistical significance level is represented by the following symbols: n.s., not significant; *P < 0.05; **P < 0.01; ***P < 0.001. Data analysis was performed using SPSS Statistics version 26.0 and Origin 2021.

## Supplementary Materials

### The PDF file includes:

Figs. S1 to S21 Tables S1 to S6 Legend for movie S1 References

## References

[1] GBD 2019 Antimicrobial Resistance Collaborators, Global mortality associated with 33 bacterial pathogens in 2019: A systematic analysis for the Global Burden of Disease Study 2019. Lancet 400, 2221–2248 (2022). 36423648 10.1016/S0140-6736(22)02185-7 PMC9763654

[2] Antimicrobial Resistance Collaborators, Global burden of bacterial antimicrobial resistance in 2019: A systematic analysis. Lancet 399, 629–655 (2022). 35065702 10.1016/S0140-6736(21)02724-0 PMC8841637

[3] P. Rudra, J. M. Boyd, Metabolic control of virulence factor production in Staphylococcus aureus. Curr. Opin. Microbiol. 55, 81–87 (2020). 32388086 10.1016/j.mib.2020.03.004 PMC7311248

[4] C. R. Belanger, A. H.-Y. Lee, D. Pletzer, B. K. Dhillon, R. Falsafi, R. E. W. Hancock, Identification of novel targets of azithromycin activity against *Pseudomonas aeruginosa* grown in physiologically relevant media. Proc. Natl. Acad. Sci. U.S.A. 117, 33519–33529 (2020). 33318204 10.1073/pnas.2007626117 PMC7777150

[5] C. L. Holmes, K. G. Dailey, K. Hullahalli, A. E. Wilcox, S. Mason, B. S. Moricz, L. V. Unverdorben, G. I. Balazs, M. K. Waldor, M. A. Bachman, Patterns of *Klebsiella pneumoniae* bacteremic dissemination from the lung. Nat. Commun. 16, 785 (2025). 39824859 10.1038/s41467-025-56095-3 PMC11742683

[6] A. A. Ordonez, M. A. Sellmyer, G. Gowrishankar, C. A. Ruiz-Bedoya, E. W. Tucker, C. J. Palestro, D. A. Hammoud, S. K. Jain, Molecular imaging of bacterial infections: Overcoming the barriers to clinical translation. Sci. Transl. Med. 11, eaax8251 (2019). 31484790 10.1126/scitranslmed.aax8251 PMC6743081

[7] L. J. Jara, G. Medina, M. A. Saavedra, Autoimmune manifestations of infections. Curr. Opin. Rheumatol. 30, 373–379 (2018). 29528865 10.1097/BOR.0000000000000505

[8] M. A. Sellmyer, I. Lee, C. Hou, C. C. Weng, S. Li, B. P. Lieberman, C. Zeng, D. A. Mankoff, R. H. Mach, Bacterial infection imaging with [^18^F]fluoropropyl-trimethoprim. Proc. Natl. Acad. Sci. U.S.A. 114, 8372–8377 (2017). 28716936 10.1073/pnas.1703109114 PMC5547613

[9] E. C. Lydon, R. Henao, T. W. Burke, M. Aydin, B. P. Nicholson, S. W. Glickman, V. G. Fowler, E. B. Quackenbush, C. B. Cairns, S. F. Kingsmore, A. K. Jaehne, E. P. Rivers, R. J. Langley, E. Petzold, E. R. Ko, M. T. McClain, G. S. Ginsburg, C. W. Woods, E. L. Tsalik, Validation of a host response test to distinguish bacterial and viral respiratory infection. EBioMedicine 48, 453–461 (2019). 31631046 10.1016/j.ebiom.2019.09.040 PMC6838360

[10] Y. Zhou, J. Dong, P. Zhao, J. Zhang, M. Zheng, J. Feng, Imaging of single bacteria with electrochemiluminescence microscopy. J. Am. Chem. Soc. 145, 8947–8953 (2023). 37040201 10.1021/jacs.2c13369

[11] J. F. Huggett, D. M. O’Sullivan, S. Cowen, M. H. Cleveland, K. Davies, K. Harris, J. Moran-Gilad, A. Winter, J. Braybrook, M. Messenger, Ensuring accuracy in the development and application of nucleic acid amplification tests (NAATs) for infectious disease. Mol. Aspects Med. 97, 101275 (2024). 38772082 10.1016/j.mam.2024.101275

[12] A. Y. Trick, J. H. Melendez, F.-E. Chen, L. Chen, A. Onzia, A. Zawedde, E. Nakku-Joloba, P. Kyambadde, E. Mande, J. Matovu, M. Atuheirwe, R. Kwizera, E. A. Gilliams, Y.-H. Hsieh, C. A. Gaydos, Y. C. Manabe, M. M. Hamill, T.-H. Wang, A portable magnetofluidic platform for detecting sexually transmitted infections and antimicrobial susceptibility. Sci. Transl. Med. 13, eabf6356 (2021). 33980576 10.1126/scitranslmed.abf6356 PMC8363034

[13] M. Z. Israr, D. Bernieh, A. Salzano, S. Cassambai, Y. Yazaki, T. Suzuki, Matrix-assisted laser desorption ionisation (MALDI) mass spectrometry (MS): Basics and clinical applications. Clin. Chem. Lab. Med. 58, 883–896 (2020). 32229653 10.1515/cclm-2019-0868

[14] T. S. Cohen, J. J. Hilliard, O. Jones-Nelson, A. E. Keller, T. O’Day, C. Tkaczyk, A. DiGiandomenico, M. Hamilton, M. Pelletier, Q. Wang, B. A. Diep, V. T. Le, L. Cheng, J. Suzich, C. K. Stover, B. R. Sellman, *Staphylococcus aureus* α toxin potentiates opportunistic bacterial lung infections. Sci. Transl. Med. 8, 329ra331 (2016). 10.1126/scitranslmed.aad9922 26962155

[15] S. Niggli, R. Kümmerli, Strain background, species frequency, and environmental conditions are important in determining *Pseudomonas aeruginosa* and *Staphylococcus aureus* population dynamics and species coexistence. Appl. Environ. Microbiol. 86, e00962-20 (2020). 32651205 10.1128/AEM.00962-20 PMC7480381

[16] O. Jones-Nelson, J. J. Hilliard, A. DiGiandomenico, P. Warrener, A. Alfaro, L. Cheng, C. K. Stover, T. S. Cohen, B. R. Sellman, The neutrophilic response to *Pseudomonas* damages the airway barrier, promoting infection by *Klebsiella pneumoniae*. Am. J. Respir. Cell Mol. Biol. 59, 745–756 (2018). 30109945 10.1165/rcmb.2018-0107OC

[17] S. A. Riquelme, D. Ahn, A. Prince, *Pseudomonas aeruginosa* and *Klebsiella pneumoniae* adaptation to innate immune clearance mechanisms in the lung. J. Innate Immun. 10, 442–454 (2018). 29617698 10.1159/000487515 PMC6785651

[18] K. H. Kim, A. Hwang, Y. Song, W. S. Lee, J. Moon, J. Jeong, N. H. Bae, Y. M. Jung, J. Jung, S. Ryu, S. J. Lee, B. G. Choi, T. Kang, K. G. Lee, 3D hierarchical nanotopography for on-site rapid capture and sensitive detection of infectious microbial pathogens. ACS Nano 15, 4777–4788 (2021). 33502164 10.1021/acsnano.0c09411

[19] J. Dong, X. Wu, Q. Hu, C. Sun, J. Li, P. Song, Y. Su, L. Zhou, An immobilization-free electrochemical biosensor based on CRISPR/Cas13a and FAM-RNA-MB for simultaneous detection of multiple pathogens. Biosens. Bioelectron. 241, 115673 (2023). 37717422 10.1016/j.bios.2023.115673

[20] D. Cai, Y. Wang, J. Zou, Z. Li, E. Huang, X. Ouyang, Z. Que, Y. Luo, Z. Chen, Y. Jiang, G. Zhang, H. Wu, D. Liu, Droplet encoding-pairing enabled multiplexed digital loop-mediated isothermal amplification for simultaneous quantitative detection of multiple pathogens. Adv. Sci. 10, e2205863 (2023). 10.1002/advs.202205863 PMC9982564 36646503

[21] W. Gu, X. Deng, M. Lee, Y. D. Sucu, S. Arevalo, D. Stryke, S. Federman, A. Gopez, K. Reyes, K. Zorn, H. Sample, G. Yu, G. Ishpuniani, B. Briggs, E. D. Chow, A. Berger, M. R. Wilson, C. Wang, E. Hsu, S. Miller, J. L. DeRisi, C. Y. Chiu, Rapid pathogen detection by metagenomic next-generation sequencing of infected body fluids. Nat. Med. 27, 115–124 (2021). 33169017 10.1038/s41591-020-1105-z PMC9020267

[22] Y. K. Cho, H. Kim, A. Bénard, H.-K. Woo, F. Czubayko, P. David, F. J. Hansen, J. I. Lee, J. H. Park, E. Schneck, G. F. Weber, I.-S. Shin, H. Lee, Electrochemiluminescence in paired signal electrode (ECLipse) enables modular and scalable biosensing. Sci. Adv. 8, eabq4022 (2022). 36129990 10.1126/sciadv.abq4022 PMC9491722

[23] S. Wang, S. Zhu, Z. Kang, Y. Chen, X. Liu, Z. Deng, K. Hu, G. Wang, Y. Zhang, G. Zang, Recent advances and future prospects of the potential-resolved strategy in ratiometric, multiplex, and multicolor electrochemiluminescence analysis. Theranostics 12, 6779–6808 (2022). 36185596 10.7150/thno.74308 PMC9516240

[24] W. Lv, H. Ye, Z. Yuan, X. Liu, X. Chen, W. Yang, Recent advances in electrochemiluminescence-based simultaneous detection of multiple targets. TrAC Trends Anal. Chem. 123, 115767 (2020).

[25] L. Yang, Q. Li, Z. Ge, C. Fan, W. Huang, DNA mechanics: From single stranded to self-assembled. Nano Lett. 24, 11768–11778 (2024). 39259830 10.1021/acs.nanolett.4c03323

[26] P. Zhan, M. J. Urban, S. Both, X. Duan, A. Kuzyk, T. Weiss, N. Liu, DNA-assembled nanoarchitectures with multiple components in regulated and coordinated motion. Sci. Adv. 5, eaax6023 (2019). 31819901 10.1126/sciadv.aax6023 PMC6884410

[27] S. Chaithongyot, N. Chomanee, K. Charngkaew, A. Udomprasert, T. Kangsamaksin, Aptamer-functionalized DNA nanosphere as a stimuli-responsive nanocarrier. Mater. Lett. 214, 72–75 (2018).

[28] Q. Jiang, S. Zhao, J. Liu, L. Song, Z. G. Wang, B. Ding, Rationally designed DNA-based nanocarriers. Adv. Drug Deliv. Rev. 147, 2–21 (2019). 30769047 10.1016/j.addr.2019.02.003

[29] Y. Zeng, R. L. Nixon, W. Liu, R. Wang, The applications of functionalized DNA nanostructures in bioimaging and cancer therapy. Biomaterials 268, 120560 (2021). 33285441 10.1016/j.biomaterials.2020.120560

[30] F. Wang, W. Li, X. Feng, D. Liu, Y. Zhang, Decoration of Pt on Cu/Co double-doped CeO(2) nanospheres and their greatly enhanced catalytic activity. Chem. Sci. 7, 1867–1873 (2016). 29899909 10.1039/c5sc04069h PMC5965059

[31] J. H. Lee, D. Y. Jo, J. W. Choung, C. H. Kim, H. C. Ham, K.-Y. Lee, Roles of noble metals (M = Ag, Au, Pd, Pt and Rh) on CeO_2_ in enhancing activity toward soot oxidation: Active oxygen species and DFT calculations. J. Hazard. Mater. 403, 124085 (2021). 33265065 10.1016/j.jhazmat.2020.124085

[32] X. Song, L. Zhao, C. Luo, X. Ren, L. Yang, Q. Wei, Peptide-based biosensor with a luminescent copper-based metal-organic framework as an electrochemiluminescence emitter for trypsin assay. Anal. Chem. 93, 9704–9710 (2021). 34242018 10.1021/acs.analchem.1c00850

[33] L. Zhao, X. Song, X. Ren, D. Fan, Q. Wei, D. Wu, Rare self-luminous mixed-valence Eu-MOF with a self-enhanced characteristic as a near-infrared fluorescent ECL probe for nondestructive immunodetection. Anal. Chem. 93, 8613–8621 (2021). 34115479 10.1021/acs.analchem.1c01531

[34] Y. Zhou, J. He, C. Zhang, J. Li, X. Fu, W. Mao, W. Li, C. Yu, Novel Ce(III)-metal organic framework with a luminescent property to fabricate an electrochemiluminescence immunosensor. ACS Appl. Mater. Interfaces 12, 338–346 (2020). 31794188 10.1021/acsami.9b19246

[35] J. J. Nogueira, F. Plasser, L. González, Electronic delocalization, charge transfer and hypochromism in the UV absorption spectrum of polyadenine unravelled by multiscale computations and quantitative wavefunction analysis. Chem. Sci. 8, 5682–5691 (2017). 28989607 10.1039/c7sc01600j PMC5621053

[36] J. Shu, Z. Han, T. Zheng, D. Du, G. Zou, H. Cui, Potential-resolved multicolor electrochemiluminescence of *N*-(4-aminobutyl)-*N*-ethylisoluminol/tetra(4-carboxyphenyl)porphyrin/TiO_2_ nanoluminophores. Anal. Chem. 89, 12636–12640 (2017). 29121769 10.1021/acs.analchem.7b04175

[37] A. R. Sekhar, Y. Chitose, J. Janoš, S. I. Dangoor, A. Ramundo, R. Satchi-Fainaro, P. Slavíček, P. Klán, R. Weinstain, Porphyrin as a versatile visible-light-activatable organic/metal hybrid photoremovable protecting group. Nat. Commun. 13, 3614 (2022). 35750661 10.1038/s41467-022-31288-2 PMC9232598

[38] Z. Yang, S. Luo, Y. Zeng, C. Shi, R. Li, Albumin-mediated biomineralization of shape-controllable and biocompatible ceria nanomaterials. ACS Appl. Mater. Interfaces 9, 6839–6848 (2017). 28150935 10.1021/acsami.6b15442

[39] Z. Yang, S. Luo, H. Li, S. Dong, J. He, H. Jiang, R. Li, X. Yang, Alendronate as a robust anchor for ceria nanoparticle surface coating: Facile binding and improved biological properties. RSC Adv. 4, 59965–59969 (2014).

[40] J. Shen, X. Zhou, Y. Shan, H. Yue, R. Huang, J. Hu, D. Xing, Sensitive detection of a bacterial pathogen using allosteric probe-initiated catalysis and CRISPR-Cas13a amplification reaction. Nat. Commun. 11, 267 (2020). 31937772 10.1038/s41467-019-14135-9 PMC6959245

[41] J. Wu, B. Tang, Y. Qiu, R. Tan, J. Liu, J. Xia, J. Zhang, J. Huang, J. Qu, J. Sun, X. Wang, H. Qu, Clinical validation of a multiplex droplet digital PCR for diagnosing suspected bloodstream infections in ICU practice: A promising diagnostic tool. Crit. Care 26, 243 (2022). 35941654 10.1186/s13054-022-04116-8 PMC9358819

[42] H. Cheng, P. Hui, J. Peng, W. Li, W. Ma, H. Wang, J. Huang, X. He, K. Wang, Enzymatic behavior regulation-based colorimetric and electrochemiluminescence sensing of phosphate using the cobalt oxyhydroxide nanosheet. Anal. Chem. 93, 6770–6778 (2021). 33885275 10.1021/acs.analchem.1c00557

[43] Y. Li, Y. Wang, Q. Wu, R. Qi, L. Li, L. Xu, H. Yuan, High-throughput fluorescence sensing array based on tetraphenylethylene derivatives for detecting and distinguishing pathogenic microbes. Spectrochim. Acta A Mol. Biomol. Spectrosc. 318, 124435 (2024). 38796890 10.1016/j.saa.2024.124435

[44] E. L. Lawrence, B. D. A. Levin, T. Boland, S. L. Y. Chang, P. A. Crozier, Atomic scale characterization of fluxional cation behavior on nanoparticle surfaces: Probing oxygen vacancy creation/annihilation at surface sites. ACS Nano 15, 2624–2634 (2021). 33507063 10.1021/acsnano.0c07584

[45] V. Baldim, F. Bedioui, N. Mignet, I. Margaill, J. F. Berret, The enzyme-like catalytic activity of cerium oxide nanoparticles and its dependency on Ce(3+) surface area concentration. Nanoscale 10, 6971–6980 (2018). 29610821 10.1039/c8nr00325d

[46] H. Dong, L. Zhang, L. Li, W. Deng, C. Hu, Z.-J. Zhao, J. Gong, Abundant Ce^3+^ ions in Au-CeO*_x_* nanosheets to enhance CO_2_ electroreduction performance. Small 15, e1900289 (2019). 30938486 10.1002/smll.201900289

[47] N. Balhara, M. Devi, A. Balda, M. Phour, A. Giri, Urine; a new promising biological fluid to act as a non-invasive biomarker for different human diseases. URINE 5, 40–52 (2023).

[48] A. Nath, S. M. Larsson, A. Lenshof, W. Qiu, T. Baasch, L. Nilsson, M. Gram, D. Ley, T. Laurell, Acoustophoresis-based blood sampling and plasma separation for potentially minimizing sampling-related blood loss. Clin. Chem. Lab. Med. 63, 2218–2225 (2025). 40613108 10.1515/cclm-2025-0539

[49] L. M. Kopcinovic, J. Culej, Pleural, peritoneal and pericardial effusions – A biochemical approach. Biochem. Med. 24, 123–137 (2014). 10.11613/BM.2014.014 PMC3936968 24627721

[50] T. T.-Q. Nguyen, E. R. Kim, M. B. Gu, A new cognate aptamer pair-based sandwich-type electrochemical biosensor for sensitive detection of *Staphylococcus aureus*. Biosens. Bioelectron. 198, 113835 (2022). 34847360 10.1016/j.bios.2021.113835

[51] X. Cao, S. Li, L. Chen, H. Ding, H. Xu, Y. Huang, J. Li, N. Liu, W. Cao, Y. Zhu, B. Shen, N. Shao, Combining use of a panel of ssDNA aptamers in the detection of *Staphylococcus aureus*. Nucleic Acids Res. 37, 4621–4628 (2009). 19498077 10.1093/nar/gkp489 PMC2724295

[52] R. Li, J. Yan, B. Feng, M. Sun, C. Ding, H. Shen, J. Zhu, S. Yu, Ultrasensitive detection of multidrug-resistant bacteria based on boric acid-functionalized fluorescent MOF@COF. ACS Appl. Mater. Interfaces 15, 18663–18671 (2023). 37036801 10.1021/acsami.3c00632

[53] Y. Sun, X. Cheng, Y. Yi, K. Quan, Q. Chen, K. Zhang, J. J. Xu, The compact integration of multiple exonuclease III-assisted cyclic amplification units for high-efficiency ratiometric electrochemiluminescence detection of MRSA. Anal. Chem. 96, 943–948 (2024). 38166359 10.1021/acs.analchem.3c05410

[54] I. Ocsoy, S. Yusufbeyoglu, V. Yılmaz, E. S. McLamore, N. Ildız, A. Ülgen, DNA aptamer functionalized gold nanostructures for molecular recognition and photothermal inactivation of methicillin-Resistant *Staphylococcus aureus*. Colloids Surf. B Biointerfaces 159, 16–22 (2017). 28778062 10.1016/j.colsurfb.2017.07.056

[55] J. Soundy, D. Day, Selection of DNA aptamers specific for live *Pseudomonas aeruginosa*. PLOS ONE 12, e0185385 (2017). 28937998 10.1371/journal.pone.0185385 PMC5609762

[56] J. Hu, K. Fu, P. W. Bohn, Whole-cell *Pseudomonas aeruginosa* localized surface plasmon resonance aptasensor. Anal. Chem. 90, 2326–2332 (2018). 29260861 10.1021/acs.analchem.7b04800 PMC5907805

[57] X. Shi, J. Zhang, F. He, A new aptamer/polyadenylated DNA interdigitated gold electrode piezoelectric sensor for rapid detection of *Pseudomonas aeruginosa*. Biosens. Bioelectron. 132, 224–229 (2019). 30877887 10.1016/j.bios.2019.02.053

[58] Z. Zhong, R. Gao, Q. Chen, L. Jia, Dual-aptamers labeled polydopamine-polyethyleneimine copolymer dots assisted engineering a fluorescence biosensor for sensitive detection of *Pseudomonas aeruginosa* in food samples. Spectrochim. Acta A Mol. Biomol. Spectrosc. 224, 117417 (2020). 31362188 10.1016/j.saa.2019.117417

[59] C. Y. Effah, L. Ding, L. Tan, S. He, X. Li, H. Yuan, Y. Li, S. Liu, T. Sun, Y. Wu, A SERS bioassay based on vancomycin-modified PEI-interlayered nanocomposite and aptamer-functionalized SERS tags for synchronous detection of *Acinetobacter baumannii* and *Klebsiella pneumoniae*. Food Chem. 423, 136242 (2023). 37196408 10.1016/j.foodchem.2023.136242

[60] A. Deb, M. Gogoi, T. K. Mandal, S. Sinha, P. S. G. Pattader, Specific instantaneous detection of *Klebsiella pneumoniae* for UTI diagnosis with a plasmonic gold nanoparticle conjugated aptasensor. ACS Appl. Bio. Mater. 6, 3309–3318 (2023). 10.1021/acsabm.3c00369 37437266

[61] S. Liu, Q. Li, H. Yang, P. Wang, X. Miao, Q. Feng, An in situ quenching electrochemiluminescence biosensor amplified with aptamer recognition-induced multi-DNA release for sensitive detection of pathogenic bacteria. Biosens. Bioelectron. 196, 113744 (2022). 34736100 10.1016/j.bios.2021.113744

[62] R. Das, A. Dhiman, A. Kapil, V. Bansal, T. K. Sharma, Aptamer-mediated colorimetric and electrochemical detection of *Pseudomonas aeruginosa* utilizing peroxidase-mimic activity of gold NanoZyme. Anal. Bioanal. Chem. 411, 1229–1238 (2019). 30637436 10.1007/s00216-018-1555-z

[63] S. Meile, J. Du, S. Staubli, S. Grossmann, H. Koliwer-Brandl, P. Piffaretti, L. Leitner, C. I. Matter, J. Baggenstos, L. Hunold, S. Milek, C. Guebeli, M. Kozomara-Hocke, V. Neumeier, A. Botteon, J. Klumpp, J. Marschall, S. McCallin, R. Zbinden, T. M. Kessler, M. J. Loessner, M. Dunne, S. Kilcher, Engineered reporter phages for detection of *Escherichia coli*, *Enterococcus*, and *Klebsiella* in urine. Nat. Commun. 14, 4336 (2023). 37474554 10.1038/s41467-023-39863-x PMC10359277

[64] H. Peng, I. A. Chen, Rapid colorimetric detection of bacterial species through the capture of gold nanoparticles by chimeric phages. ACS Nano 13, 1244–1252 (2019). 30586498 10.1021/acsnano.8b06395 PMC6396317

[65] M. Imai, K. Mine, H. Tomonari, J. Uchiyama, S. Matuzaki, Y. Niko, S. Hadano, S. Watanabe, Dark-field microscopic detection of bacteria using bacteriophage-immobilized SiO_2_@AuNP core-shell nanoparticles. Anal. Chem. 91, 12352–12357 (2019). 31464422 10.1021/acs.analchem.9b02715

[66] P. Liu, Y. Wang, L. Han, Y. Cai, H. Ren, T. Ma, X. Li, V. A. Petrenko, A. Liu, Colorimetric assay of bacterial pathogens based on Co_3_O_4_ magnetic nanozymes conjugated with specific fusion phage proteins and magnetophoretic chromatography. ACS Appl. Mater. Interfaces 12, 9090–9097 (2020). 32023032 10.1021/acsami.9b23101

[67] B. Duncan, N. D. B. Le, C. Alexander, A. Gupta, G. Y. Tonga, M. Yazdani, R. F. Landis, L.-S. Wang, B. Yan, S. Burmaoglu, X. Li, V. M. Rotello, Sensing by smell: Nanoparticle-enzyme sensors for rapid and sensitive detection of bacteria with olfactory output. ACS Nano 11, 5339–5343 (2017). 28423269 10.1021/acsnano.7b00822 PMC5848077

[68] D. Svechkarev, M. R. Sadykov, K. W. Bayles, A. M. Mohs, Ratiometric fluorescent sensor array as a versatile tool for bacterial pathogen identification and analysis. ACS Sens. 3, 700–708 (2018). 29504753 10.1021/acssensors.8b00025 PMC5938749

[69] A. Suea-Ngam, P. D. Howes, A. J. deMello, An amplification-free ultra-sensitive electrochemical CRISPR/Cas biosensor for drug-resistant bacteria detection. Chem. Sci. 12, 12733–12743 (2021). 34703560 10.1039/d1sc02197d PMC8494034

[70] J. Li, W. Shen, X. Liang, S. Zheng, Q. Yu, C. Wang, C. Wang, B. Gu, 2D film-like magnetic SERS tag with enhanced capture and detection abilities for immunochromatographic diagnosis of multiple bacteria. Small 20, e2310014 (2024). 38193262 10.1002/smll.202310014

[71] J. Li, Z. Li, B. Wang, Q. Yu, T. Wu, C. Wang, B. Gu, Electropositive magnetic fluorescent nanoprobe-mediated immunochromatographic assay for the ultrasensitive and simultaneous detection of bacteria. Adv. Sci. 12, e2412421 (2025). 10.1002/advs.202412421 PMC11948010 39804983

[72] E. A. Idelevich, U. Reischl, K. Becker, New microbiological techniques in the diagnosis of bloodstream infections. Dtsch. Arztebl. Int. 115, 822–832 (2018). 30678752 10.3238/arztebl.2018.0822 PMC6369238

[73] G. Jannes, D. De Vos, in *Diagnostic Bacteriology Protocols*, L. O’Connor, Ed. (Humana Press, 2006), pp. 1–21.

[74] D. Wang, S. Wang, X. Du, Q. He, Y. Liu, Z. Wang, K. Feng, Y. Li, Y. Deng, ddPCR surpasses classical qPCR technology in quantitating bacteria and fungi in the environment. Mol. Ecol. Resour. 22, 2587–2598 (2022). 35587727 10.1111/1755-0998.13644

[75] M. J. Espy, J. R. Uhl, L. M. Sloan, S. P. Buckwalter, M. F. Jones, E. A. Vetter, J. D. C. Yao, N. L. Wengenack, J. E. Rosenblatt, F. R. Cockerill III, T. F. Smith, Real-time PCR in clinical microbiology: Applications for routine laboratory testing. Clin. Microbiol. Rev. 19, 165–256 (2006). 16418529 10.1128/CMR.19.1.165-256.2006 PMC1360278

[76] Y. Peng, R. Xie, Y. Luo, P. Guo, Z. Wu, Y. Chen, P. Liu, J. Deng, B. Huang, K. Liao, Clinical evaluation of a multiplex droplet digital PCR for diagnosing suspected bloodstream infections: A prospective study. Front. Cell. Infect. Microbiol. 14, 1489792 (2024). 39885964 10.3389/fcimb.2024.1489792 PMC11779721

[77] A. van Belkum, M. Welker, M. Erhard, S. Chatellier, Biomedical mass spectrometry in today’s and tomorrow’s clinical microbiology laboratories. J. Clin. Microbiol. 50, 1513–1517 (2012). 22357505 10.1128/JCM.00420-12 PMC3347093

