# Supplementary material for: DNA-directed electrochemiluminescence nanosphere with electrocatalysis-enhanced microfluidic arrays for rapid multibacterial detection
Source: Sci Adv. 2025 Dec 12;11(50):eady3070. doi: 10.1126/sciadv.ady3070 (PMC12700205; doi:10.1126/sciadv.ady3070)
Supplement: Supplementary file 1 — Figs. S1 to S21 Tables S1 to S6 Legend for movie S1 References [file sciadv.ady3070_sm.pdf]

Science Advances
AAAS

Supplementary Materials for

DNA-directed electrochemiluminescence nanosphere with electrocatalysis-enhanced microfluidic arrays for rapid multibacterial detection

Chengli Zhang *et al.*

Corresponding author: Yuhui Liao, liaoyh8@mail.sysu.edu.cn; Yu Fu, hellfuyu@163.com;
Jiajian Zhou, zhoujj2013@gmail.com

Sci. Adv. **11**, eady3070 (2025)
DOI: 10.1126/sciadv.ady3070

The PDF file includes:

Figs. S1 to S21
Tables S1 to S6
Legend for movie S1
References

Other Supplementary Material for this manuscript includes the following:

Movie S1

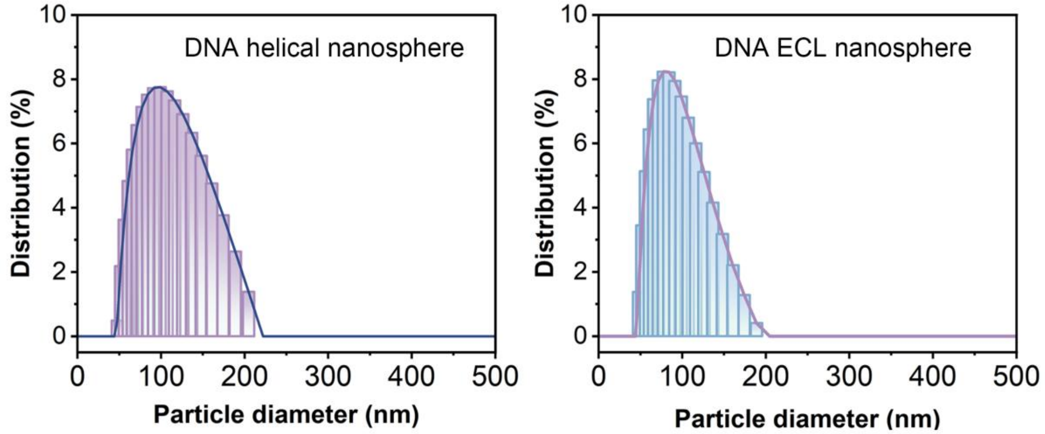

Fig. S1. Dynamic light scattering results of DNA helical and DNA ECL nanosphere.

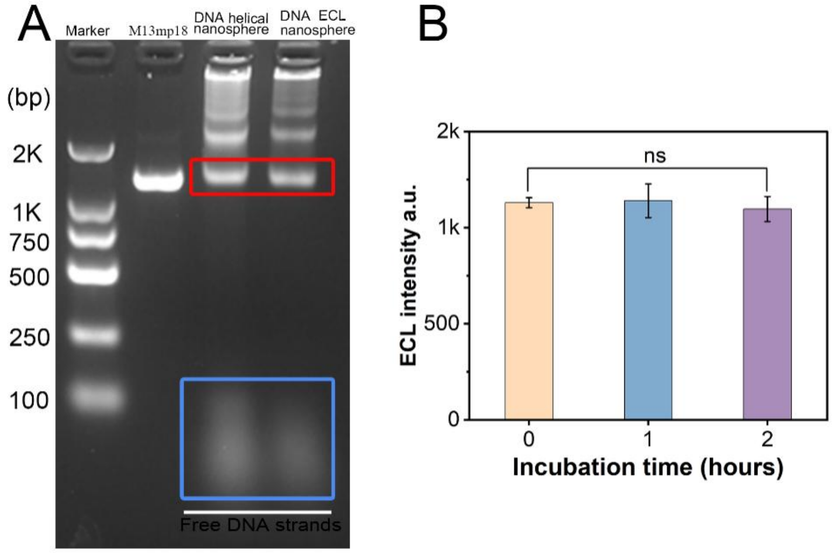

Fig. S2. Verification of the stability of DNA ECL nanosphere 37°C. (**A**) Electropherogram obtained after 2 hours of incubation at 37°C using agarose gel electrophoresis. (**B**) ECL results of DNA ECL nanosphere obtained after 2 hours of incubation at 37°C. The experiments were repeated for three times (n = 3) and data were presented as mean ± sd. ns = not significant.

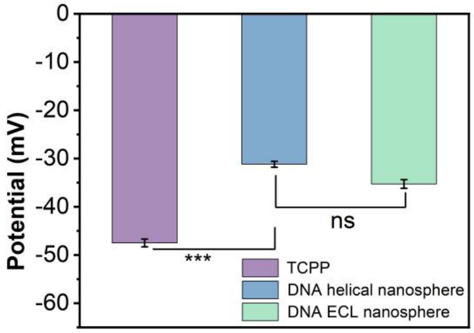

Fig. S3. The result of ζ-potential. The experiments were repeated for three times (n = 3) and data were presented as mean ± sd. *p < 0.05, **p < 0.01, ***p < 0.001, ns = not significant.

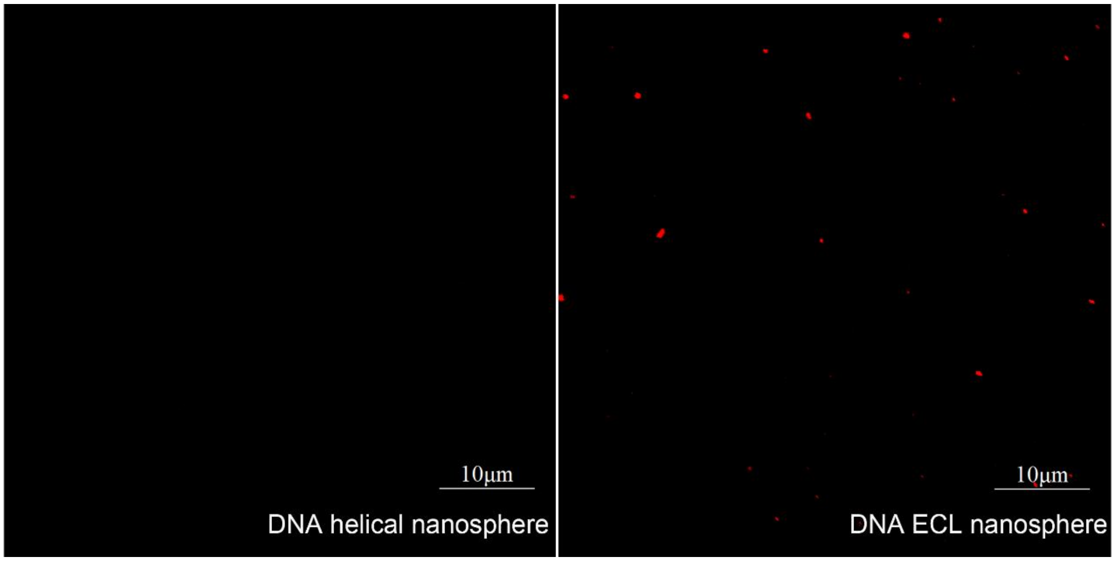

Fig. S4. Characterization of DNA helical nanosphere and DNA ECL nanosphere. Confocal laser scanning microscopy images of DNA helical nanosphere and DNA ECL nanosphere.

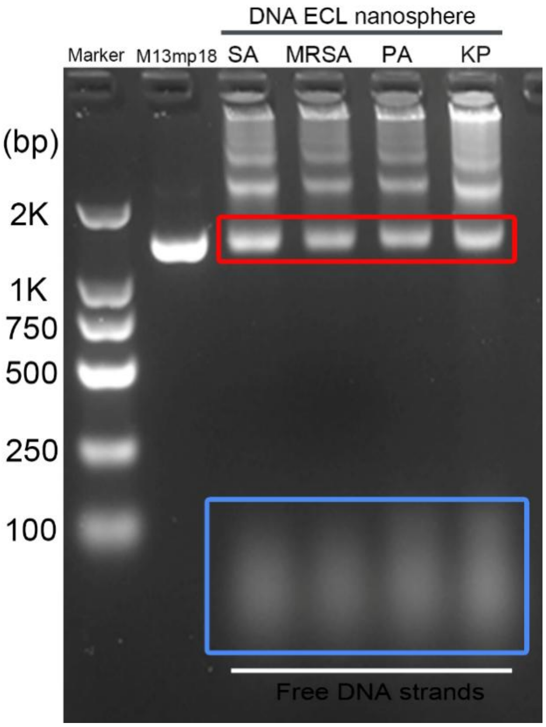

Fig. S5. Electropherogram (agarose gel electrophoresis). Identification of DNA ECL nanosphere electropherograms for different bacterial pathogens. ^{SA}*S. aureus*, ^{PA}*P. aeruginosa* and ^{KP}*K.pneumoniae.*

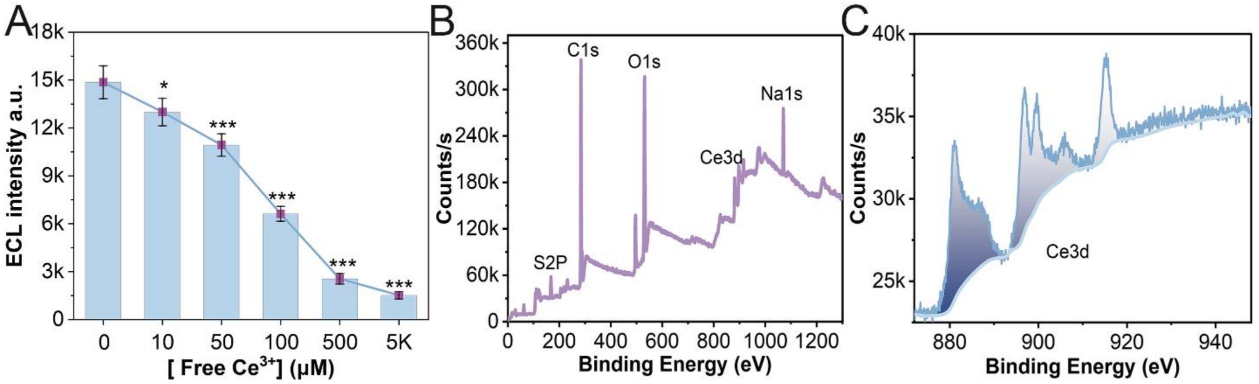

Fig. S6. Verification of the effect of free Ce^{3+} and X-ray photoelectron spectroscopy (XPS) results. (**A**) ECL responses of free Ce^{3+} at various concentrations with $S_2O_8^{2-}$-TCPP (1.2 μg/mL). Statistical analysis comparing various concentration of free Ce^{3+} vs. free Ce^{3+} (0 μM). (**B**) XPS survey scan of NanoCe. (**C**) Ce 3d XPS spectrum of NanoCe. The experiments were repeated for three times (n = 3) and data were presented as mean ± sd. *p < 0.05, **p < 0.01, ***p < 0.001, ns = not significant.

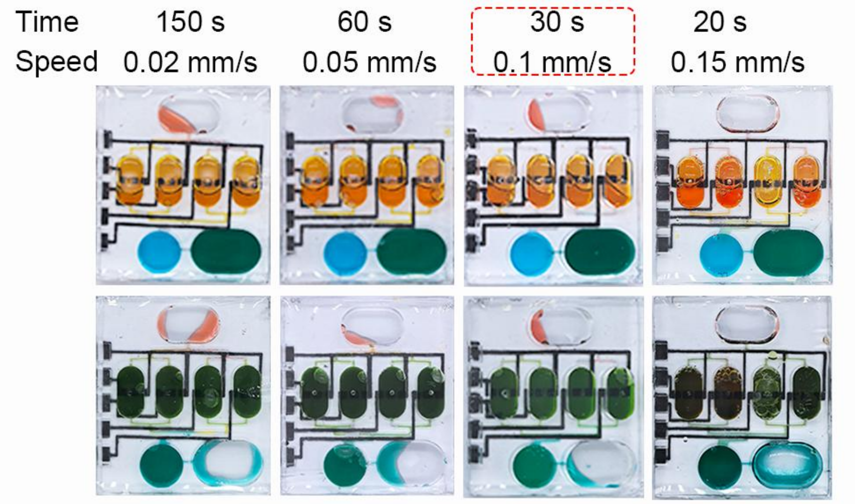

Fig. S7. Optimization of mixing condition. The Pressing speed of stepper motor pressure.

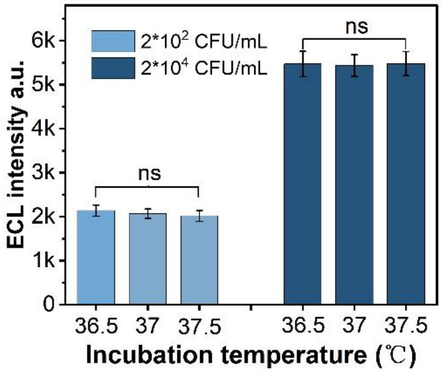

Fig. S8. Verification of the effect of temperature fluctuations on detection performance. The experiments were repeated for three times (n = 3) and data were presented as mean ± sd. ns = not significant.

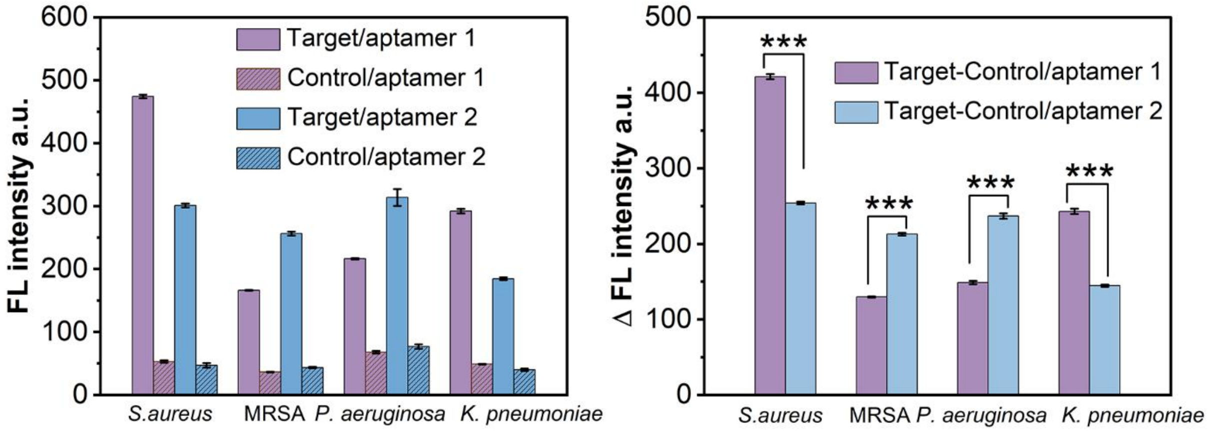

Fig. S9. Verification of specific binding of bacterial pathogens to the corresponding aptamers. Fluorescence spectra illustrating the specific binding interactions between bacterial pathogens and their corresponding aptamers. Δ Fluorescence a.u.=Δ Fluorescence a.u. (Target) **-** Δ Fluorescence a.u. (Control). The experiments were repeated for three times ($n = 3$) and data were presented as mean ± sd. *$p < 0.05$; **$p < 0.01$, ***$p < 0.001$, ns = not significant.

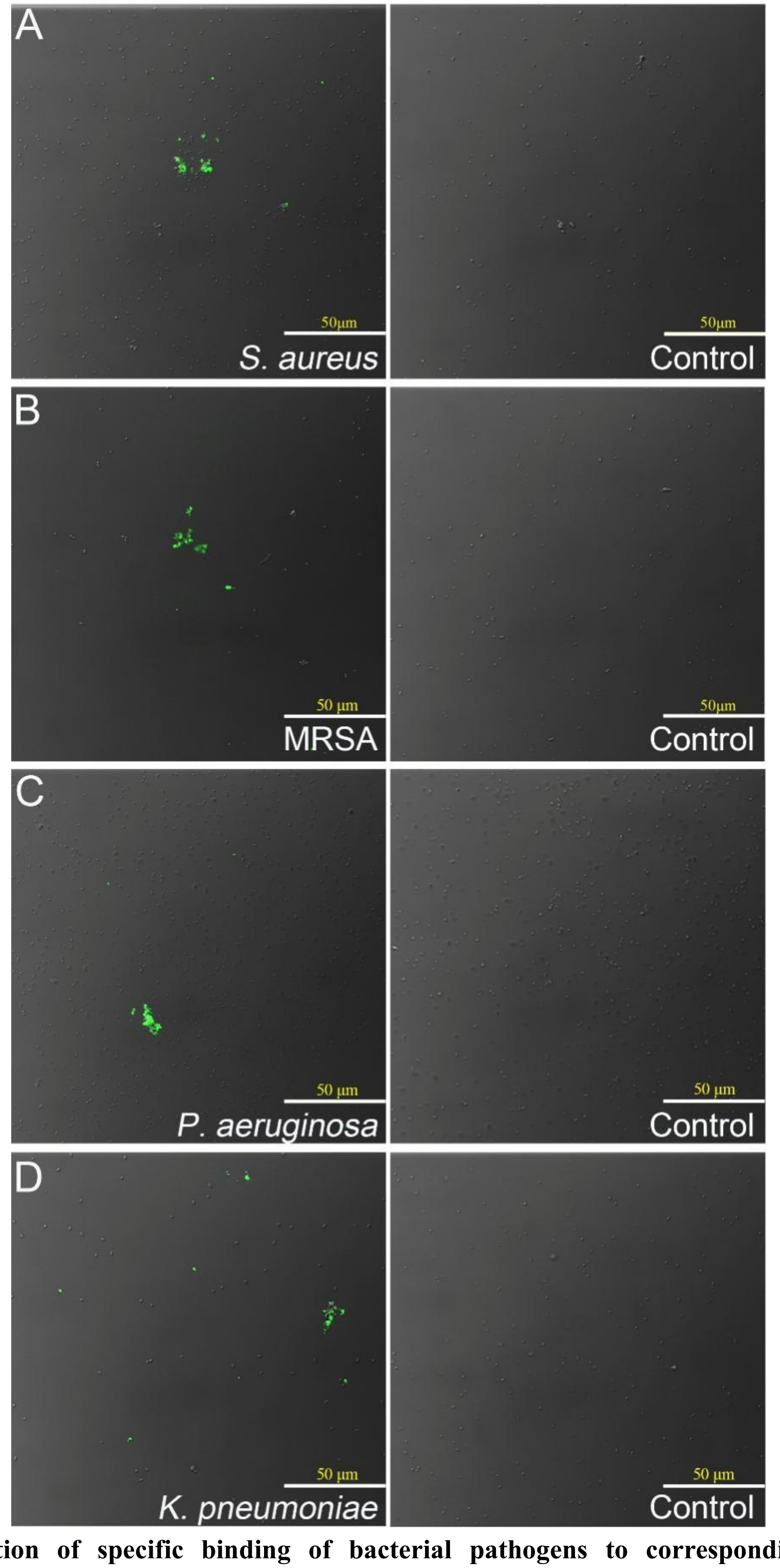

Fig. S10. Verification of specific binding of bacterial pathogens to corresponding aptamers. (**A**) Fluorescence images of *S. aureus* and control. (**B**) Fluorescence images of MRSA and control. (**C**) Fluorescence images of *p. aeruginosa* and control. (**D**) Fluorescence images of *K. pneumoniae* and control.

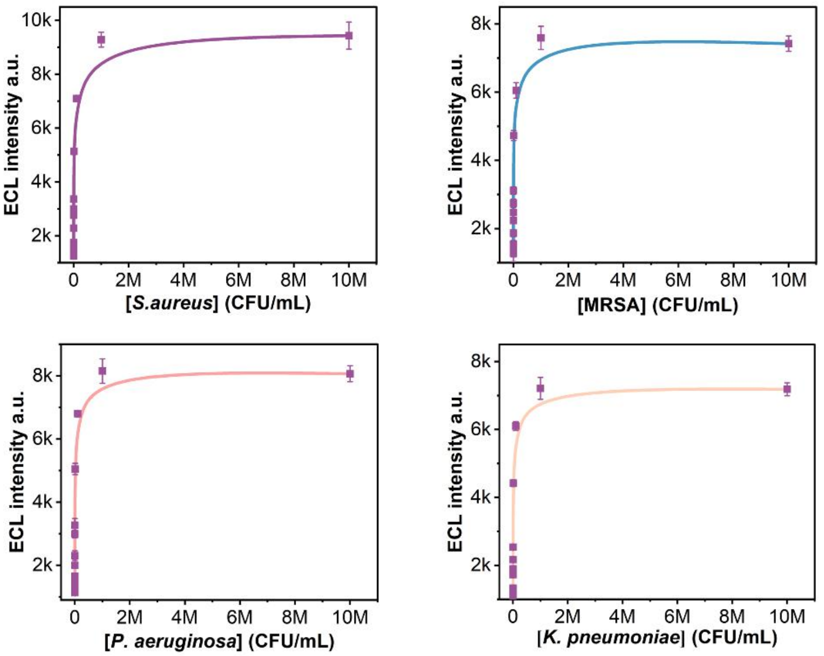

Fig. S11. Performance of the ECL microfluidic arrays for multiple types of bacterial analyses. ECL responses of the ECL microfluidic arrays to different concentrations of *S. aureus*, MRSA, *P. aeruginosa*, and *K. pneumoniae*. The experiments were repeated for three times (n = 3) and data were presented as mean ± sd.

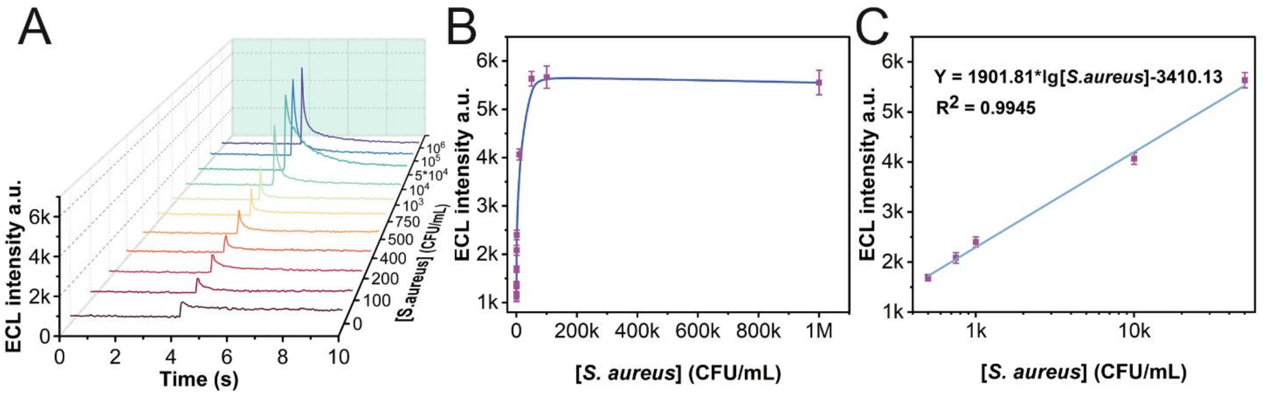

Fig. S12. Performance of the $S_2O_8^{2-}$-TCPP ECL sensor for *S.aureus* analyses. (**A**) and (**B**) ECL responses of $S_2O_8^{2-}$-TCPP ECL sensor towards different concentrations of *S. aureus*. (**C**) Linear analysis of the ECL detection results. The experiments were repeated for three times (n = 3) and data were presented as mean ± sd.

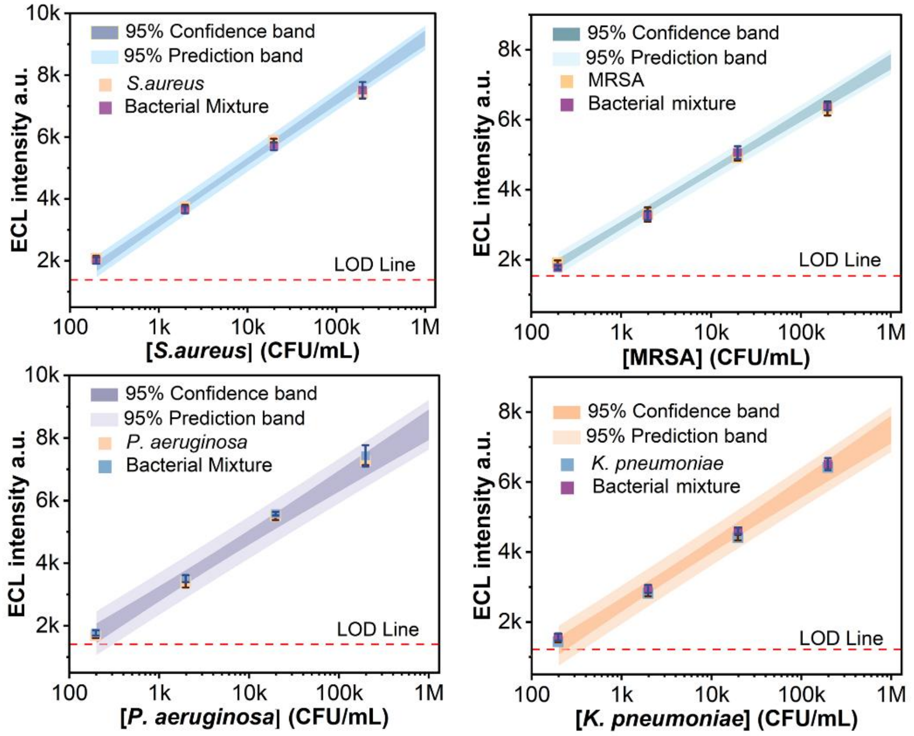

Fig. S13. Performance of the ECL microfluidic arrays for multiple types of bacterial analyses. Selectivity of the ECL microfluidic arrays. Bacterial mixture: Non-target bacteria concentrations were 10 times higher than the target bacteria. The experiments were repeated for three times (n = 3) and data were presented as mean ± sd.

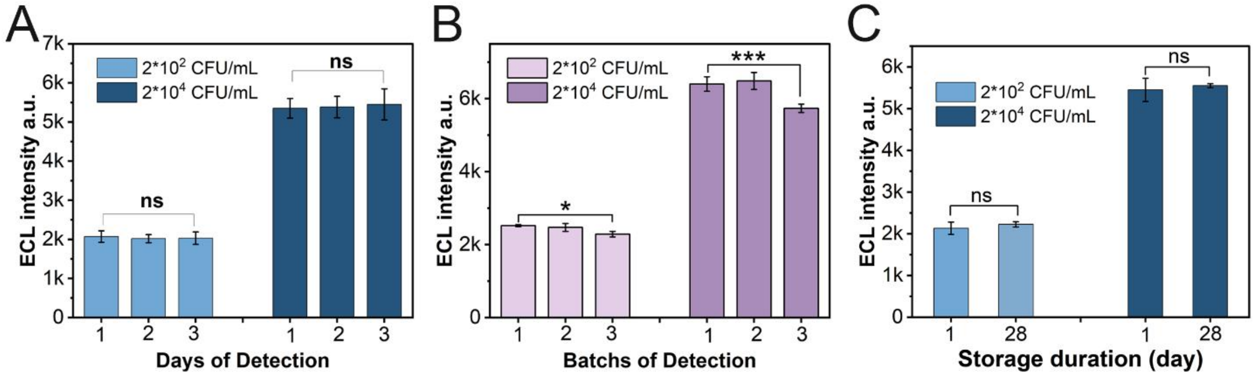

Fig. S14. Performance of the ECL microfluidic arrays. (**A**) The intra-batch reproducibility of DNA ECL nanosphere as detection reagent. (**B**) The inter-batch reproducibility of DNA ECL nanosphere as detection reagent (**C**) The stability of DNA ECL nanosphere as detection reagent. The experiments were repeated for three times (n = 3) and data were presented as mean ± sd. *p < 0.05, **p < 0.01, ***p < 0.001, ns = not significant.

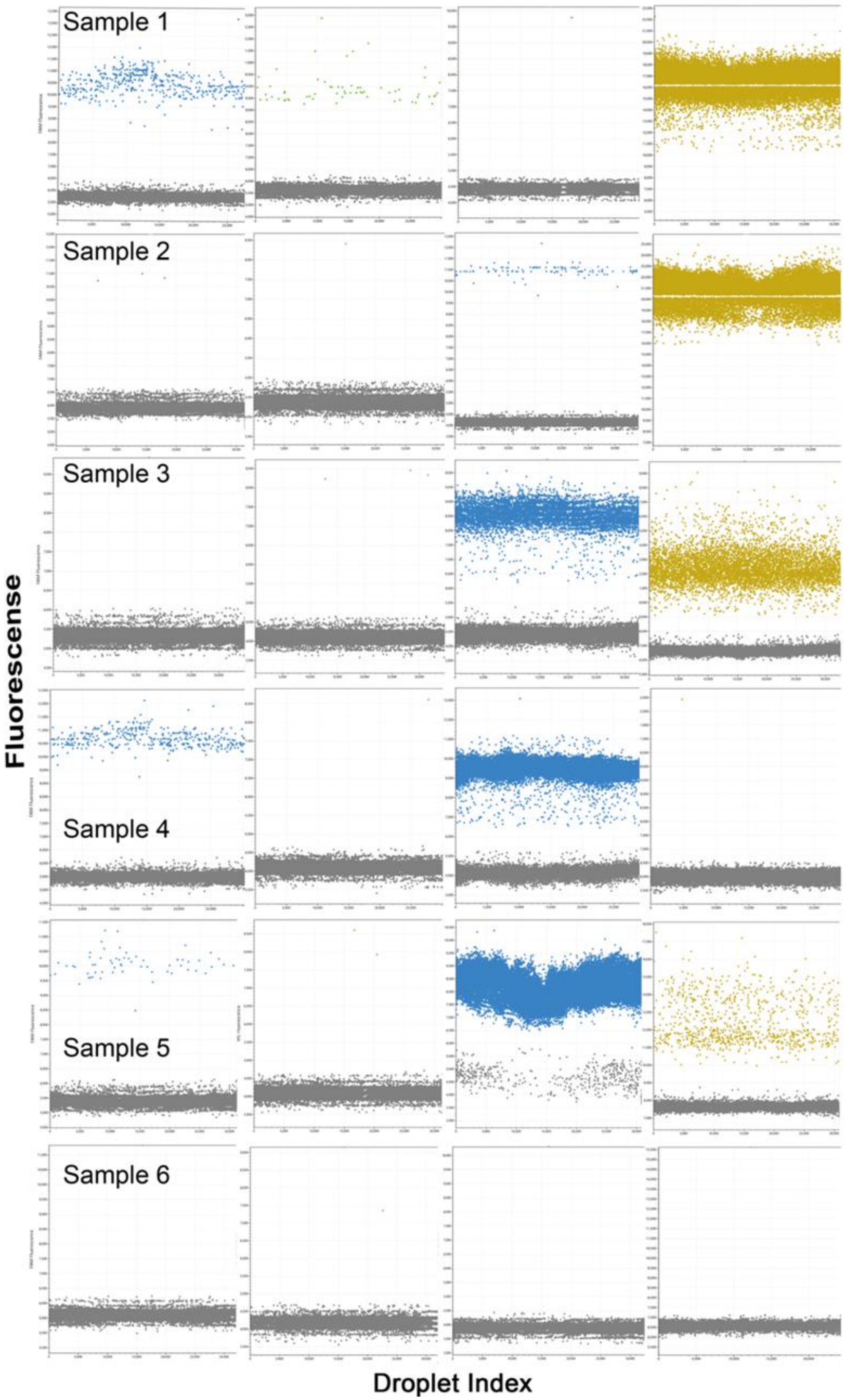

Fig. S15. Digital PCR analysis of 6 urine samples. The order of digital PCR results from left to right is *S. aureus*, MRSA, *P. aeruginosa*, and *K. pneumoniae.*

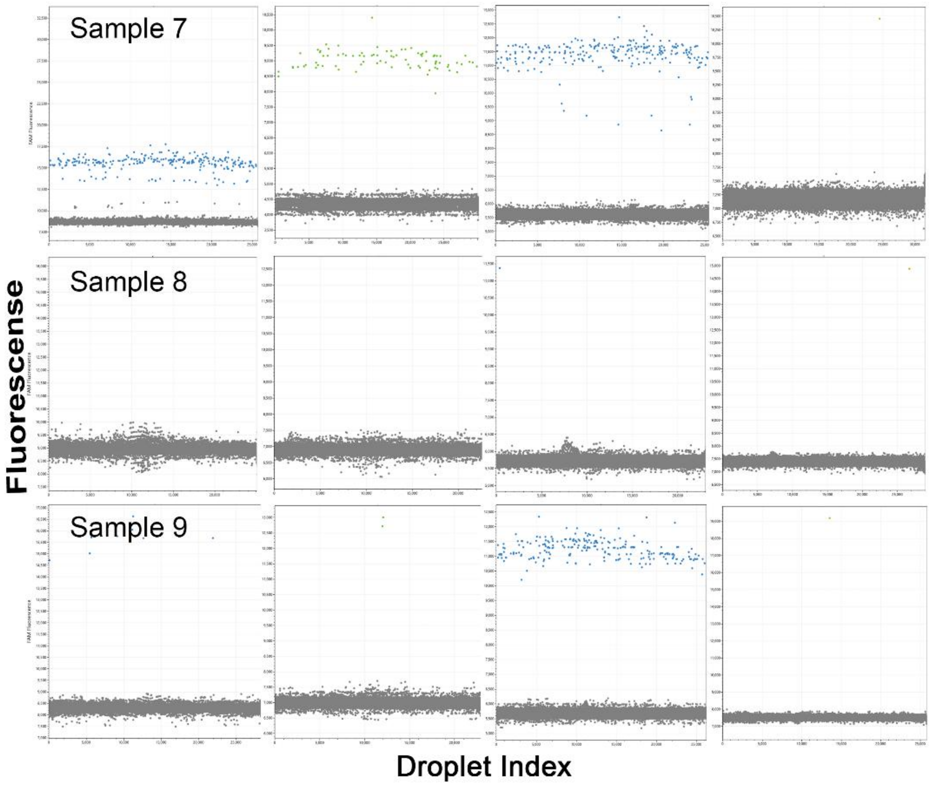

Fig. S16. Digital PCR analysis of 3 urine samples. The order of digital PCR results from left to right is *S. aureus*, MRSA, *P. aeruginosa*, and *K. pneumoniae.*

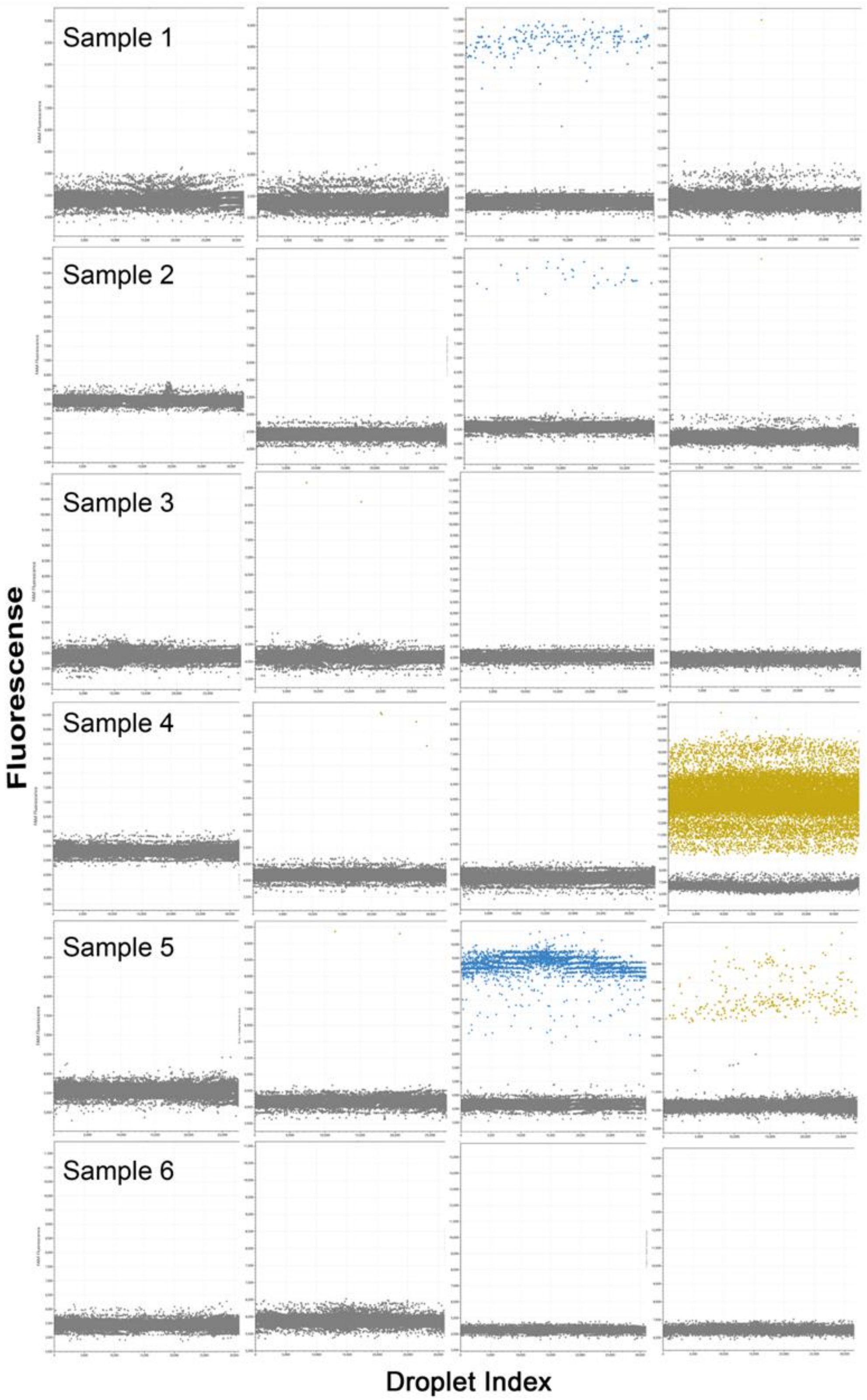

Fig. S17. Digital PCR analysis of 6 pleuroperitoneal effusion samples. The order of digital PCR results from left to right is *S. aureus*, MRSA, *P. aeruginosa*, and *K. pneumoniae.*

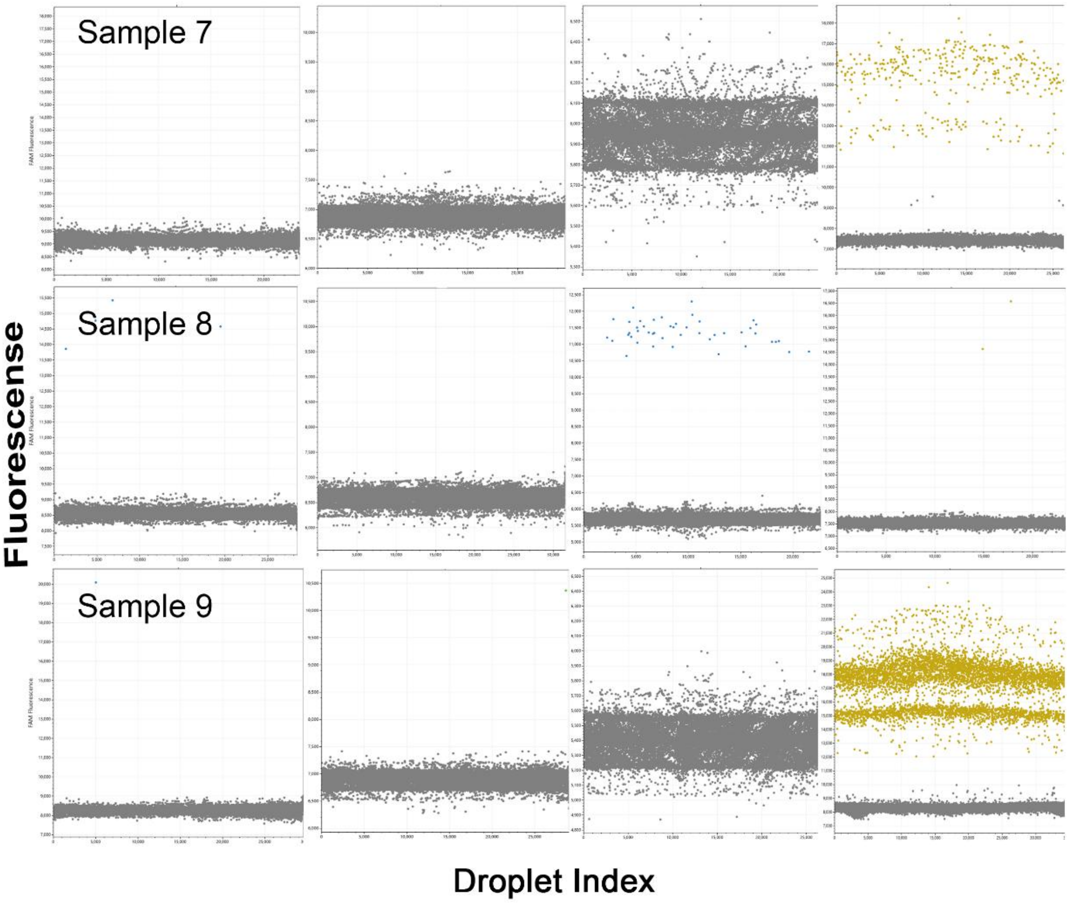

Fig. S18. Digital PCR analysis of 3 pleuroperitoneal effusion samples. The order of digital PCR results from left to right is *S. aureus*, MRSA, *P. aeruginosa*, and *K. pneumoniae.*

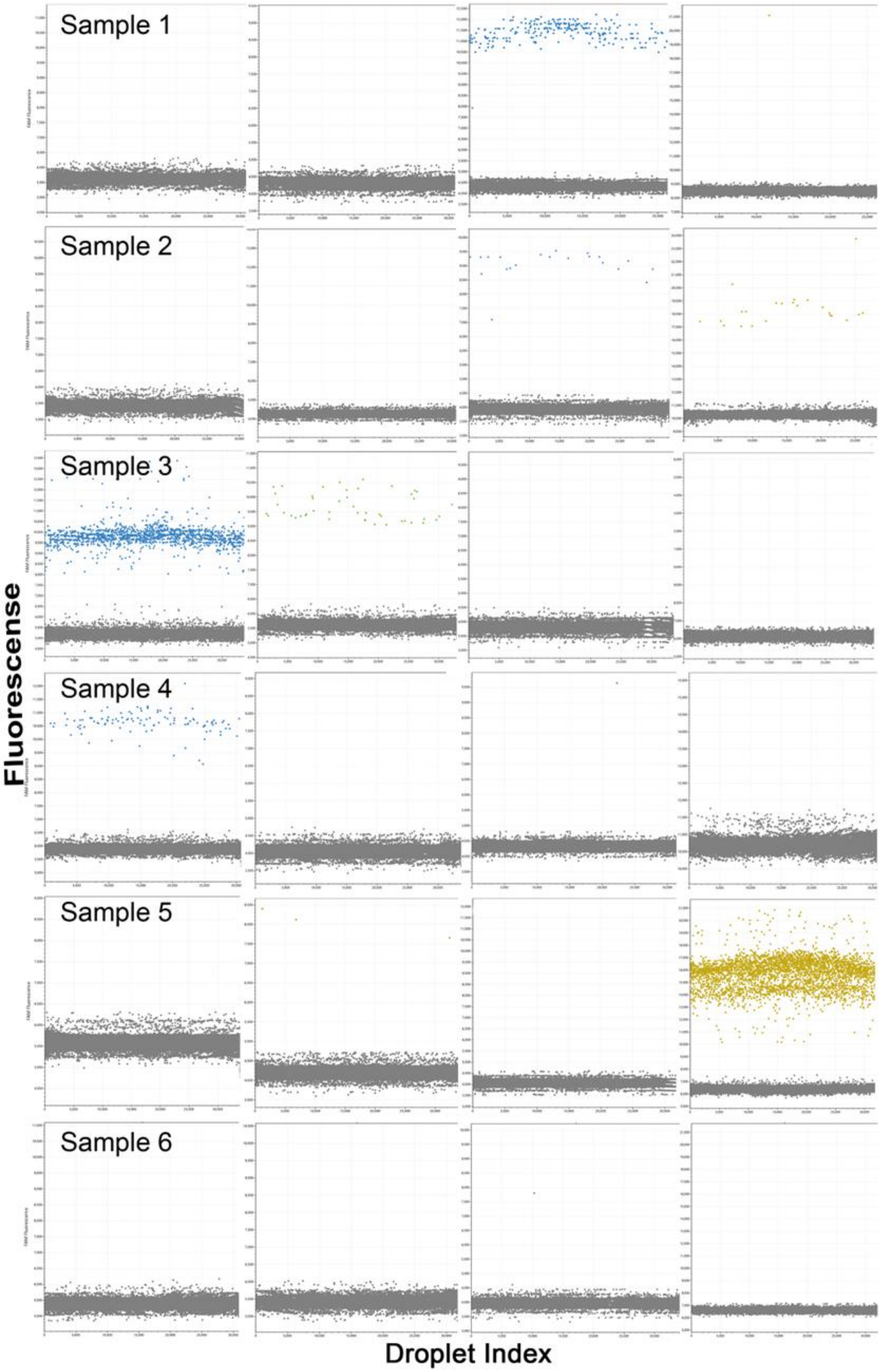

Fig. S19. Digital PCR analysis of 6 blood samples. The order of digital PCR results from left to right is *S. aureus*, MRSA, *P. aeruginosa*, and *K. pneumoniae*.

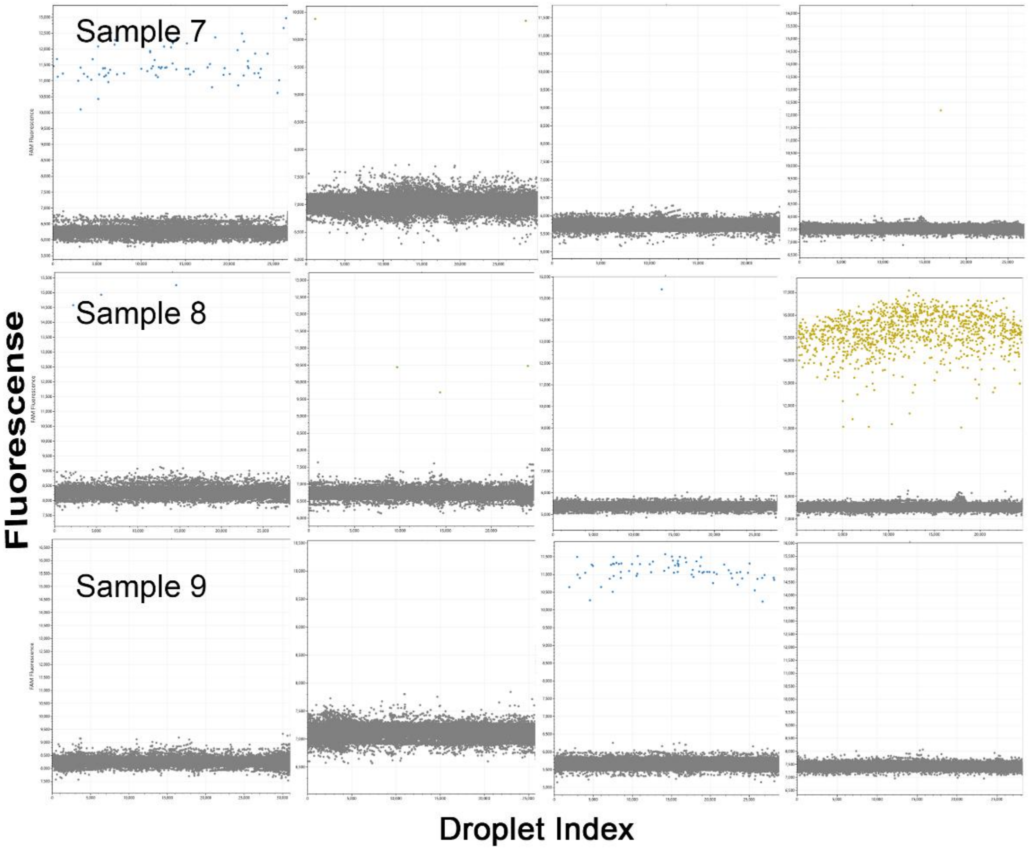

Fig. S20. Digital PCR analysis of 3 blood samples. The order of digital PCR results from left to right a is *S. aureus*, MRSA, *P. aeruginosa*, and *K. pneumoniae*.

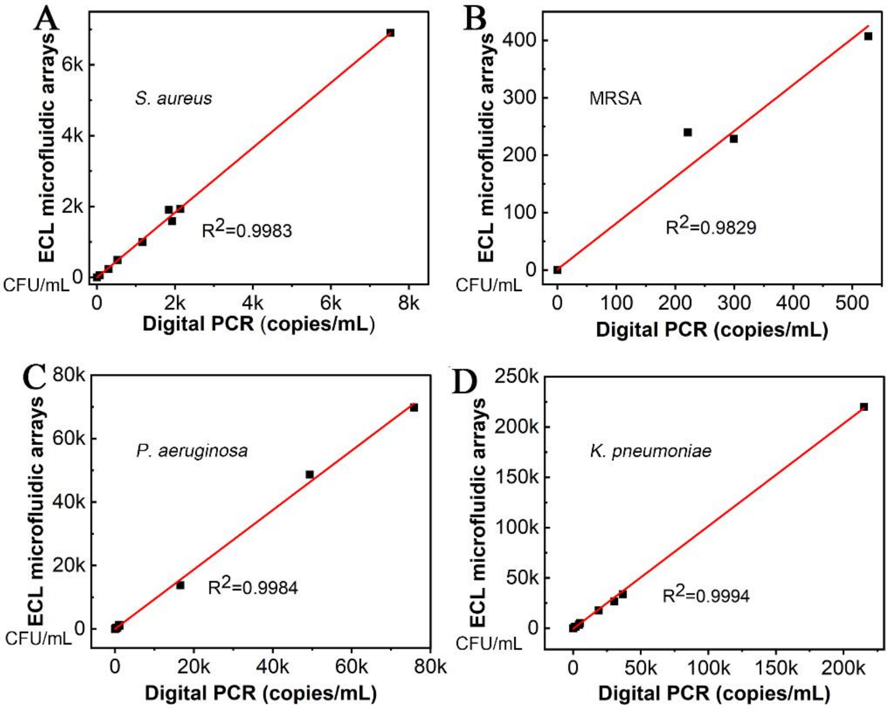

Fig. S21. Regression analysis of digital PCR results and ECL results. (**A**) Regression analysis of *S. aureus.* (**B**) Regression analysis of MRSA. (**C**) Regression analysis of *P. aeruginosa.* (**D**) Regression analysis of *K. pneumoniae.*

Table S1. The DNA strands for the DNA helical nanosphere.

Name	Sequence 5'→3'
	166 fixed short DNA strands
**a-1**	TTCCATTAAATTAAAGGGATTTTAGACAGGGAGATAGAAC
**a-2**	CCTTCTGACCCAGCCCTCATAGTTAGCGTAATGAGGAAGT
**a-3**	CAGGGCGATGGAGCTAAACAGGAGGCCGACGGGTAAAAT
**a-4**	ACGTAATGCCATGGTTTAATTTCAACTTTAACCGTCTAT
**a-5**	AATGAATCGGCCAACGTGGACTCCAACGTCA
**a-6**	AAGGGCGAAAAATCATTGTGATCGTGCCAGCTGCATT
**a-7**	CCAACCTAAAACGGCTTTGAGGACTAAAGACTTTTTCACGATCTAAAGTT
**a-8**	TAAGAACTGGCTAACGAGTAGTAAATTGGGCTTGAGACTACGAAGGCA
**a-9**	ATAAATATCGGGAAACCTGATTACCTTATGCGATTT
**a-10**	GCGAAAGAGACGAGAACACCAGCATTATCTGCGGAATCGTC
**a-11**	TTGTCGTCTTTCCAGACGTTAGTAAATGAGCAACGGCTACAGAGAAAGAG
**a-12**	ACTGCCCGCTTTCCAGTTCATTGAATCCC
**a-13**	CCTCAAATGCTTTGCTCCTTTTGATAAGAGGGGATGTGCTGCAAGG
**a-14**	CGATTAAGTTGGGTAATTAATTGCGTTGCGCTC
**a-15**	TGCATCTGCCTACGCCAGCTGGCGAAAGGGTCATTTTTGC
**a-16**	GGATGGCTTATAGATACATTCATCGTAACCG
**a-17**	CAATTCTAGATGGGCGTCGCAAATGGTGGCAT
**a-18**	TCATCTTTGACGCGAAAGACAGCATCGGAACGAGGGTAATTTTC
**a-19**	GGAAGAAAAATCTATTCAGTGAATAAGGCTTGCCCTATACACTAAAACAC
**a-20**	TGGATAGCGTCCAATAACCAGTCAGGACGTTG
**a-21**	GATTATACTAACAAAGCTGCTCACGTTAAGTAAAATGTTTAGAC
**a-22**	ATTTTGCTAAACAACTTTCAACAGTTTCGTCACCCTCAGCACCCCAGC
**a-23**	AATAGAAAGGAACAACTAAAGGAATTGCGCTGAGGCTTGCAGAGATTTGT
**a-24**	ATCATCGCTTCGACAAGAACCACAGGTTTTTGCAAAAGAAGTT
**a-25**	TTGCCAGAGGGTAAATCAAAAATCAGGTCTTTACCCTGACCAGACCGGAA
**a-26**	GCAAACTCCAACAGATCTGGTGCTGTAGCTCAACATGTTTTTCTGCGA
**a-27**	ACGAGTAGACCTGTTTAGCTATATGCTGAAAAGGTCAATAATTTAGTT
**a-28**	TGACCATGAGCTTAATTGCTGAGTCAGGATTAGAG
**a-29**	AGTACCTTTAATTAAACAGTTCAGAA
**a-30**	AACGAGAATGACCAGGTAATATAAAACGAACTAACG
**a-31**	GAACAACATTATTGGATATTCATTACCCAAATCAACGCAAGCGCGAAAC
**a-32**	AAAGTACAACGGGGAGTTAAAGGCCGCTTTTGCGGGATCAGCGGAGTGAG
**a-33**	TGACAACAACCATCGCGACCTG
**a-34**	CTCCATGTCCAGGCGCATAGGCACCACAAGACGACGATAAAAAC
**a-35**	CAAAATAGCGAGAGGCAGAAAGATTCATCAGTTG
**a-36**	AGATTTAGGAATTGGCTGACCTTCATCAAGAGTAATCCTGATAAATTGTGTCG
**a-37**	AAATCCGCCCACGCATAACCGATATATTCGGTCGAATAATAATTTTTTCACGTTG AAA
**a-38**	AGTAATAAAAGGGACATTCTGGCCAACAAACGGTACGCCAGAAGTTGGAA
**a-39**	TCAGAGCGGGCCCACTACGTGAAGAACGCGCGGGGAGAGGCGGT
**a-40**	TTGCGTATTGGGGGGTGCCTAATGAGTGAGCTAACTCACACGCCAGGGTT
**a-41**	TTCCCAGTCACGACGGCGATCGGTGCGGGCCTCTTCGCTATAGTTTGAG
**a-42**	GGACGACATAGGTTACGTTGGTGTACTAATAGTATGGGGACCGTAT
**a-43**	CGGCCTCACAACTGTTGGGAAGGTTGTAAAACGAC
**a-44**	GGCCAGTGCCAAGACGAGCCGGAAGCAT
**a-45**	AAAGTGTAAAGCCTGCGCCATCCAGTTTGGAACAAGA
**a-46**	GTCCACTATTAAACCATCACCCAAATCAAGTTTTTGAGCACGTATAAC

**a-47**	GTGCTTTCCTCTCTTGAGAAGTGTTTTTATAATCAGTCAGTCACACGACC
**a-48**	CATTGGCAGATTCACGAGGCCACCGAGTAATATGGTT
**a-49**	GCTTTGACTGGGGTCGAGGTGCTGTTGTGGGTGGTTTTTCTTTT
**a-50**	CACCAGTGAGACGGGCTCAAAAGAATAGCCC
**a-51**	GAGATAGGGTTGAGCGTAAAGCACTAAATCGGAACCCAATGCGCCGCTA
**a-52**	CAGGGCGCGTACAAGAGTCTGTCCATCACGCAAATTACTCAATCGTCTGAAATGG
**a-53**	TACCTACATTTTGACGACCGTTGTAGCAATAACACCCG
**a-54**	CCGCGCTTTAAAGGGAGCCCCCTATAAAAACAGCTGATTGCCCT
**a-55**	TCACCGCCTGGTGTTATCCGCTCACAATTCCACACAACATCTTGCATGCC
**a-56**	TGCAGGTCGACTCTAAGCGCCATTCGC
**a-57**	CATTCAGGCTGCGGGAAGATCGCACTCCCGGCGGAT
**a-58**	TGACCGTAAGTAGCATTTCGGATTGCGCGATTTCAACAGTTGA
**a-59**	TTCCCAATAAATATGCAACTAATTTTAATTCGAGC
**a-60**	TTCAAAGCGAACTATTATAGTCAGAAG
**a-61**	CAAAGCGGATTGCATTTACCTTCAACTAATGCAGAT
**a-62**	ACATAACGCCAAAAGGACAGATGAACGGTGTACAGATACTTAGCCGGAAC
**a-63**	GAGGCGCAGACCTTGATACCGATAGTTGCGCCGACAA
**a-64**	CCTTTAATTGTATCGGTTTATCAGCTTGCTTTCTGGTAATA
**a-65**	TGCCTGAGTAGAAGAGGCGCTG
**a-66**	GCAAGTGTCGGCGAACGTGGCGCTGTTTGAGAGTTGCAGCAAGC
**a-67**	GGTCCACGCTGGCATAGTAAGAGCAACACT
**a-68**	ATCATAACCCTCGTCAAAAAGATTAAG
**a-69**	AGGAAGCCCGTAATCATGGTCATAGCTGT
**a-70**	TTCCTGTGTGAAATCCCTGAGATGGTGGTTCCGAAATC
**a-71**	GGCAAAATCCCTGATTTAGAGCTTGACGGGGAAAGCAGCGGTCACGCTGCGCG
**a-72**	TAACCACCCTTCTTTGATTAGTAATAACATCACTGCAACAGGAAAAACGCTCATG GAAA
**a-73**	TCGGCCTTGCGAGGTGAATTTCTTAAACAGGGTCAATCAT
**a-74**	AAGGGAACCGCGAAAGGAGCGGGCGCTAGACTCAAACTA
**a-75**	GGGAAGAAAGAACTGACCAACTTTGAAAGAGGAATTACGAGGTTTGCCCCA
**a-76**	GCAGGCGAAAATCAGAAAGGAA
**a-77**	GGTACCGAGCTCGAATTCGAAAGACTTCAAATATCGCGAGTACGGTGT
**a-78**	CTGGAAGTTTTCTGGTGCCGGAAACCAGGCAAGAGGATCCCCG
**a-79**	CGGCACCGCTCATTCCATGTATTTGGGCTCCGTG
**a-80**	GGAACAAAAGCCAGCTTTC
**b-1**	TTATCATTTTACAGTGCCCGTATAAACAGTAACGCCTGTA
**b-2**	GCATTCCACAAAAGCGTAAGAATACGTGGCTTGAGTAACA
**b-3**	CCATCTTTTCGGGGTCAGTGCCTTGAGTAGCGGAACAAA
**b-4**	GAAACCACCACAATATATGTGAGTGAATATTAGCGTTTG
**b-5**	AAATTCATATGGTTTAGGCATTTTCGGTCAT
**b-6**	AGCCCCCTTAACCTTGCTTCTTGTCACAATCAATAGA
**b-7**	TTATCATCATATCCCGAACGTTATTAATTTTAAAAGTACAGACAATATTT
**b-8**	ATTAATTTTCCCTTTTAATGGAAACAGTACATAAATGAAGGAGCGGAA
**b-9**	AAGCCTGTTAAGTTTATTTGTAAATCGTCGCTATTA
**b-10**	TATCAGATATTTGAATTACCTTTTAGAATAATTACTAGAAA
**b-11**	TTGAATGGCTATTAGTCTTTAATGCGCGGTATTAAATCCTTTGTCCTGAT
**b-12**	GCAAAGACACCACGGAATTAGTATCATAT
**b-13**	GCGTTATACAAAAACAATAGATAAGTCCTGATAATTGAGCGCTAAT
**b-14**	ATCAGAGAGATAACCCCAACATATAAAAGAAAC
**b-15**	TTTCCAGAGCCCCTGAACAAAGTCAGAGGGACAAGAAAAA

**b-16**	TAATATCCCATTACCGCGCCTAACGAGCGTC
**b-17**	AACCTCTTACCAACGCCAATAGCAAGCTAGCG
**b-18**	AATATAATCCTAGACTTTACAAACAATTCGACAACTCAACTGATAGCCCT
**b-19**	GATAGCTTAGATTAATTAATTACATTTAACAATTTCGATGGCAATTCATC
**b-20**	ATAAACACCGGAATCATCCTTGAAAACATAGC
**b-21**	TGGATTATCATCAAGAAAACAAAAGACGAAGGCGTTAAATAAGA
**b-22**	GGATTTAGAAGTATTGATTGTT
**b-23**	AGATAAAACAGAGGTGAGGCGGTCAGTCAACTAATAGATTAGAACCAACC
**b-24**	ATATCAAATTTCAATTACCTGAATCATTTAATGGTTTGAAATA
**b-25**	CCGACCGTGTGCAGTAGGGCTTAATTGAGAATCGCCATATGACGACAATA
**b-26**	AACAACATGTTCAGATGTAGAAACCAATCAATAATCGGCTCAAGCCGT
**b-27**	TTTTATTTAGATATAGAAGGCTTAGCGAGGCGTTTAAATCTCATCGTA
**b-28**	GGAATCATCCTAATTTACGAGCCTAATGCAGAACG
**b-29**	CGCCTGTTTATCTTCTTACCAGTATA
**b-30**	AAGCCAACGCTCAAATAAATCTGAGAAGAGTCAATA
**b-31**	GTGAATTTATCAAAGCAAAAGAAGATGATGAAACAAAACTTCTGAATTA
**b-32**	TGGAAGGAATTGAGCCGTCAATAGATAATACATTTGAAACCACCAGCAGA
**b-33**	GCTGAGAGCCAGCAGAATTGAGGAAGGTTATAAAGAA
**b-34**	ATTGCGTAAATACCAAGTTACAGCTTAGTATTTTAGTTAATTTC
**b-35**	ATCTTCTGACCTAAATAGGTCTGAGAGACTACCT
**b-36**	TTTTAACCTCCGAAATCGCGCAGAGGCGAATTATTCAATTATTAGCAC
**b-37**	GTAAAACAGAAATCTAAAATATCTTTAGGTGCACTAAATTAACACCGCCTGCAAC
**b-38**	ACTGAGTTTCGTCACCAGTACAAACTACTAATGCCCCCTGCCTGGTAATA
**b-39**	AGTTTTAACATAATCAAAATCATTCATCCCAGCGCCAAAGACAA
**b-40**	AAGGGCGACATAAACGTAGAAAATACATACATAAAGGTGGACAAGAATTG
**b-41**	AGTTAAGCCCAATAACATTAGACGGGAGAATTAACTGAACACTAATTTG
**b-42**	CCAGTTACAATTTTATCCTGAATCCCGACTTGCGGCTACAAAATAAA
**b-43**	CAGCCATAAAAAACAGGGAAGCGTAAGAGCAAGAA
**b-44**	ACAATGAAATAGCTAAGACTCCTTATTA
**b-45**	CGCAGTATGTTAGCTCAACCTTGCCTTTAGCGTCAGA
**b-46**	CTGTAGCGCGTTCCGGAACCAGAGCCACCACCGGACTTTTGATGATAC
**b-47**	AGGAGTGTACTATTTCGGAACCTATTATTCTGAAACACATGTACCGTAAC
**b-48**	TGAAAGTATTAAGAGAAGCGTC
**b-49**	ATACATGGACCGCCTCCCTCAGTCAAGTGATTGAGGGAGGGAAG
**b-50**	GTAAATATTGACGGAATAGCAGCACCGTAAT
**b-51**	CAGTAGCGACAGAAAGCCGCCACCCTCAGAACCGCCAAGTCTCTGAATT
**b-52**	TACCGTTCCAGTGCTGAGACTCCTCAAGAGAAGGATTCACCACCCTCATT
**b-53**	TTCAGGGATAGCA
**b-54**	CCACCCTCAGAACCGCCACCCTCAGAGCAGGATTAGCGGGGTTCAGAATG
**b-55**	GAAAGCGCCCCTCAGAGCCACCCATCGAATTATTCATTAAAGGT
**b-56**	GAATTATCACCAACGGAATACCCAAAAGAACTGGCATGATAATAGCTATC
**b-57**	TTACCGAAGCCCTTAGCAGCCTTTACA
**b-58**	GAGAGAATAACATTTATTTATCCCAATCTGCTATTT
**b-59**	TGCACCCAGGAGGTTTTGAAGCCTAAGAACTCCGGCTCATCGA
**b-60**	GAACAAGGTCTTTCCTTATCATAAAGGTAAAGTAA
**b-61**	TTCTGTCCAGACTTAACAACGCCAACA
**b-62**	TGTAATTTAGGCAGCAAATAGTTGGGTTATATAACT
**b-63**	ATATGTAAATGCTAACGGATTCGCCTGATTGCTTTGGATTTTCAGGTTTA
**b-64**	ACGTCAGATGACAGTTGGCAAATCAACAGTAGAAAGGCAAATGAAAAATC
**b-65**	TAAAGCATCACCTT

**b-66**	CGTACTCAGGAGGTTTAGTACCGCCCGTCGAGAGGGTTGATGGCCTT
**b-67**	GATATTCAACCACCAGAGCCGCTACCATGACTTGAGCCATTTGG
**b-68**	GAATTAGAGCCATCGCAAGACAAAGAACGC
**b-69**	GAGAAAACTTTTTAGGCATTTTCGAGC
**b-70**	CAGTAATAAAGTTACCAGAAGGAAACCGA
**b-71**	GGAAACGCAATAATGTCACCTAGCAAGGCCGGAAACGT
**b-72**	CACCAATGAAACACCCTCAGAGCCGCCACCAGAACCCAAACGAATGGATC
**b-73**	TTCATTAAAGCTTGCTCAGTACCAGGCGGATAAGTGCACCCTCAGAACCG
**b-74**	GCCCGGAATAACCCTCAATCAATATCTGGTATATACAGTA
**b-75**	ACAGTACCTTTTGAGGCAGGTCAGACGATTATAAGTATA
**b-76**	TGACAGGAGGTTACATCGGGAGAAACAATGATGCAAATCCAAGCAAAATCA
**b-77**	CCAGTAGCACCATCGCCAGCAT
**b-78**	AAGCAGATAGCCGAACAAGAGAATATAAAGTACCGACATCCAAGAACG
**b-79**	GGTATTAAACTTTAACGTCAAAAATGAAAATTTTAAGAAAAGT
**b-80**	CGATTTTTTGCAAGTACCGCATATTCTTAAATCA
**b-81**	AGATTAGTCAAATAAGAAA
**a-lock-2**	ATTATTTATTAAAAAAAAAAAAAAAAAAAAA
**a-lock-4**	TGTATGGGTTAAAAAAAAAAAAAAAAAAAAA
**a-lock-5**	GCTCCAAAAGGAGTTAAAAAAAAAAAAAAAAAAAAA
**b-lock-1**	AGTGCCACTTAAAAAAAAAAAAAAAAAAAAA
**b-lock-3**	TGTATCACTTAAAAAAAAAAAAAAAAAAAAA

5 variable short DNA strands and aptamer strands	
***S. aureus* lock-aptamer**	TTTTTTTTTTT**GCAATGGTA**CGGTACTTCCTCGGCACGTTCTCAGTAGCGCTCGCTGGTCATCCCACAGCTACGTCAAAAGTGCACGCTACTT**TGCTAA**TTTTTTTTTTTTTTTT
***S. aureus* a-lock-1**	AAA**TTAGCATACCATTGC**AAATTCAGAACAATATTACCGCCAGCCATT
***S. aureus* a-lock-3**	AAA**TTAGCATACCATTGC**AAATTATCTCCAAAAAAAAG
***S. aureus* b-lock-2**	AAA**TTAGCATACCATTGC**AAATTAACATCGCCATTAAAAATACCGAACG
***S. aureus* b-lock-4**	AAA**TTAGCATACCATTGC**AAATTAGCCCAATAGGAACC
***S. aureus* b-lock-5**	AAA**TTAGCATACCATTGC**AAATTGCTGAACCTCAAATATCAAGG
**MRSA lock-aptamer**	TTTTTTTTTTT**ATTGGTCAT**GCGGTTGGTTGCGGTTGGGCATGATGTATT**TCTGTG**TTTTTTTTTTTTTTTT
**MRSA a-lock-1**	AAA**CACAGAATGACCAAT**AAATTCAGAACAATATTACCGCCAGCCATT
**MRSA a-lock-3**	AAA**CACAGAATGACCAAT**AAATTATCTCCAAAAAAAAG
**MRSA b-lock-2**	AAA**CACAGAATGACCAAT**AAATTAACATCGCCATTAAAAATACCGAACG
**MRSA b-lock-4**	AAA**CACAGAATGACCAAT**AAATTAGCCCAATAGGAACC
**MRSA b-lock-5**	AAA**CACAGAATGACCAAT**AAATTGCTGAACCTCAAATATCAAGG
***P. aeruginosa* lock-aptamer**	TTTTTTTTTTT**ATCCCCCGT**TGCTTTCGCTTTTCCTTTCGCTTTTGTTCGTTTCGTCCCTGCTTCCT**TTCTTG**TTTTTTTTTTTTTTTT
***P. aeruginosa* a-lock-1**	AAA**CAAGAAACGGGGGAT**AAATTCAGAACAATATTACCGCCAGCCATT
***P. aeruginosa* a-lock-3**	AAA**CAAGAAACGGGGGAT**AAATTATCTCCAAAAAAAAG
***P. aeruginosa* b-lock-2**	AAA**CAAGAAACGGGGGAT**AAATTAACATCGCCATTAAAAATACCGAACG
***P. aeruginosa* b-lock-4**	AAA**CAAGAAACGGGGGAT**AAATTAGCCCAATAGGAACC

***P. aeruginosa* b-lock-5**	AAACAAGAAACGGGGGATAAATTGCTGAACCTCAAATATCAAGG
***K. pneumoniae* lock-aptamer**	TTTTTTTTTTTCCTATAATCAGGCTCAGCATGGAGTTGCGAGGCCAATATCCG GTTAAGCGTTTTTTTTTTTTTTTT
***K. pneumoniae* a-lock-1**	AAACGCTTAGATTATAGGAAATTCAGAACAATATTACCGCCAGCCATT
***K. pneumoniae* a-lock-3**	AAACGCTTAGATTATAGGAAATTATCTCCAAAAAAAAG
***K. pneumoniae* b-lock-2**	AAACGCTTAGATTATAGGAAATTAACATCGCCATTAAAAATACCGAACG
***K. pneumoniae* b-lock-4**	AAACGCTTAGATTATAGGAAATTAGCCCAATAGGAACC
***K. pneumoniae* b-lock-5**	AAACGCTTAGATTATAGGAAATTGCTGAACCTCAAATATCAAGG

The above DNA strands are non-universal sequences synthesized to target different bacteria. The lock strands labeled with the same color indicate that they form complementary connections through base pairing with their corresponding aptamers in the DNA nanosphere structure.

Table S2. 6-FAM-labeled bacterial aptamer sequences.

Name	Sequence 6 FAM labeled 5'→3'
S. aureus **aptamer 1** *(51)*	GCAATGGTACGGTACTTCCTCGGCACGTTCTCAGTAGCGCTCGCTG GTCATCCCACAGCTACGTCAAAAGTGCACGCTACTTTGCTAA
S. aureus **aptamer 2** *(50)*	GCTTCCAGCTTATTGAATAAAGACGGGGGGGGGGACCGGCGTATG AGTGAAGATGGGGGCGCTGAAGCGCGGAAGC
**MRSA aptamer 1**(*52*)	CCATCCACACTCCGCAAGGGTGCCCCGGGGGGCTGTTCAGCGTGGT GGTGGGATGCCGTTTTGGTCCTTAGTCTCCGTCGTCGGCTGCCTCTA CAT
**MRSA aptamer 2** (*53, 54*)	ATGCGGTTGGTTGCGGTTGGGCATGATGTATTTCTGTG
P. aeruginosa **aptamer 1** *(55)*	AGAGCGTCGGTGTGGTAACTGTTCAGGAGGATGACATTGTCGCCT
P. aeruginosa **aptamer 2** *(56-58)*	CCCCCGTTGCTTTCGCTTTTCCTTTCGCTTTTGTTCGTTTCGTCCCTG CTTCCTTTCTTG
K. pneumoniae **aptamer 1** *(59)*	AATCAGGCTCAGCATGGAGTTGCGAGGCCAATATCCGGTTAAGCG
K. pneumoniae **aptamer 2** *(60)*	GGCTGGATGGGGCGTGTGGAGCCCCGTTAGAATATCAGAGGTGGT GGCAACGGTGCGGACAGCG

Table S3. The spiking method for verifying bacteria and aptamer binding.

Name	Target	Non-specific bacterial mixtures (Control)
***S. aureus* aptamer 1**	*S. aureus*	*A. baumannii*, MRSA, *P. aeruginosa*, *K. pneumoniae* and *E. coli*
***S. aureus* aptamer 2**	*S. aureus*	*A. baumannii*, MRSA, *P. aeruginosa*, *K. pneumoniae* and *E. coli*
**MRSA aptamer 1**	MRSA	*A. baumannii*, *S. aureus*, *P. aeruginosa*, *K. pneumoniae* and *E. coli*
**MRSA aptamer 2**	MRSA	*A. baumannii*, *S. aureus*, *P. aeruginosa*, *K. pneumoniae* and *E. coli*
***P. aeruginosa* aptamer 1**	*P. aeruginosa*	*A. baumannii*, *S. aureus*, MRSA, *K. pneumoniae* and *E. coli*
***P. aeruginosa* aptamer 2**	*P. aeruginosa*	*A. baumannii*, *S. aureus*, MRSA, *K. pneumoniae* and *E. coli*
***K. pneumoniae* aptamer 1**	*K. pneumoniae*	*A. baumannii*, *S. aureus*, MRSA, *P. aeruginosa* and *E. coli*
***K. pneumoniae* aptamer 2**	*K. pneumoniae*	*A. baumannii*, *S. aureus*, MRSA, *P. aeruginosa* and *E. coli*

Note: *A. baumannii*: *Acinetobacter baumannii*, *E. coli*: *Escherichia coli*.

Table S4. Comparison of performance parameters of the ECL sensor with other bacterial detection methods.

Method	Bacteria species	LOD (CFU/mL)	Linear range (CFU/mL)	Wash	Pipetting frequency (times)	Time	Ref
In situ quenching ECL biosensor	*S. aureus*	3	10 to 10^7	√	4	＞3h	(*61*)
Colorimetric and electrochemical detection	*P. aeruginosa*	60	60 to 6.0×10^7	×	2	10 min	(*62*)
Engineered reporter phages and luminescence#	*E. coli, Klebsiella spp*., and Enterococcus *spp*	<10^2	-	×	>6	<5 h	(*63*)
AuNP aggregation and colorimetric detection#	*E. coli*, *P. aeruginosa*, *V. cholerae*, *X. campestri*s	10^2	-	√	8	>60 min	(*64*)
Dark-field microscopy imaging#	*S. aureus*	$8*10^4$	-	×	2	15-20 min	(*65*)
Magnetophoretic chromatography and colorimetric assay	*S. aureus*	8	10 to 10^4	√	4	>1 h	(*66*)
Olfactory output-nanoparticle-enzyme sensors#	Does not distinguish between bacterial species	10^2	-	×	4	45 min	(*67*)
A ratiometric fluorescent sensor array#	*S. aureus*, *S. epidermidi*s, *Bacillus subtilis*, *Enterococcus faecalis*, *E. col*i, *A. baumanni*i, *K. pneumoniae*, *Citrobacter freundii*	-	-	√	16	30 min	(*68*)
Electrochemical CRISPR/Cas biosensor	MRSA mecA gene	3.5 fM	10 fM to 0.1 nM	√	4	90 min	(*69*)
SERS–immunoc-hromatography platform	*S. aureus*, *P. aeruginos*a, *Salmonella typhimurium*	10	10 to 10^5	√	3	30 min	(*70*)
Magnetic fluorescent nanoprobe-mediated immunochromatographic assay	*P. aeruginos*a, *S. pneumoniae*, *S. typhimurium*	8 – 40 cells/mL	-	√	2	>15min	(*71*)
The $S_2O_8^{2-}$-TCPP ECL sensor	*S. aureus*	450	500 to $5*10^4$	×	1	45 min	This work
This sensor	*S. aureus*, MRSA, *K. pneumoniae*, and *P. aeruginos*a	≤100	100/200 to 10^6	×	1	45 min	This work

#Qualitative detection method. Adding frequency: reagent or sample adding frequency.

Table S5. Comparison of performance parameters of the ECL sensor with other traditional detection methods.

Method	LOD	Linear range	Time	Ref
Bacterial culture and identification	10 CFU/mL	—	24-72 hours	(*72, 73*)
qPCR	1 or 10 copies/μL	10 to 10^8 or 1 to 10^7 copies/μL	2-3 hours	(*74, 75*)
Digital PCR	50 copies/mL	1 to 10^5 copies/mL	3-5 hours	(*41, 76*)
MALDI-TOF MS	10^5 CFU	Qualitative testing	>16 hours	(*77*)
This sensor	≤100 CFU/mL	100/200 to 10^6 CFU/mL	45 min	This work

Table S6. Spiking method to validate the specificity of ECL microfluidic arrays.

**Target**	**Various concentration of target (CFU/mL)**	**Bacterial mixture**	**Correct identification Yes/No**
S. aureus	$2*10^2$, $2*10^3$, $2*10^4$, $2*10^5$	*S. aureus*, *A. baumannii*, MRSA, *P. aeruginosa*, *K. pneumoniae*, *E. coli, S. epidermidis*, *P. fluorescens*, and *K. acidifaciens*	yes
**MRSA**		MRSA, *A. baumannii*, *S. aureus*, *P. aeruginosa*, *K. pneumoniae*, *E. coli, S. epidermidis*, *P. fluorescens*, and *K. acidifaciens*	yes
P. aeruginosa		*P. aeruginosa, A. baumannii*, *S. aureus*, MRSA, *K. pneumoniae*, E. coli, *S. epidermidis*, *P. fluorescens*, and *K. acidifaciens*	yes
K. pneumoniae		*K. Pneumoniae, A. baumannii*, *S. aureus*, MRSA, *P. aeruginosa*, *E. coli, S. epidermidis*, *P. fluorescens*, and *K. acidifaciens*	yes

Concentrations of non-target bacteria were 10 times higher than target bacteria.

Other Supplementary Materials for this manuscript include the following:

Movie S1 (.mp4 format). The operating video of the ECL microfluidic detection.

REFERENCES AND NOTES

1. GBD 2019 Antimicrobial Resistance Collaborators, Global mortality associated with 33 bacterial pathogens in 2019: A systematic analysis for the Global Burden of Disease Study 2019. *Lancet* **400**, 2221–2248 (2022).

2. Antimicrobial Resistance Collaborators, Global burden of bacterial antimicrobial resistance in 2019: A systematic analysis. *Lancet* **399**, 629–655 (2022).

3. P. Rudra, J. M. Boyd, Metabolic control of virulence factor production in Staphylococcus aureus. *Curr. Opin. Microbiol.* **55**, 81–87 (2020).

4. C. R. Belanger, A. H.-Y. Lee, D. Pletzer, B. K. Dhillon, R. Falsafi, R. E. W. Hancock, Identification of novel targets of azithromycin activity against *Pseudomonas aeruginosa* grown in physiologically relevant media. *Proc. Natl. Acad. Sci. U.S.A.* **117**, 33519–33529 (2020).

5. C. L. Holmes, K. G. Dailey, K. Hullahalli, A. E. Wilcox, S. Mason, B. S. Moricz, L. V. Unverdorben, G. I. Balazs, M. K. Waldor, M. A. Bachman, Patterns of *Klebsiella pneumoniae* bacteremic dissemination from the lung. *Nat. Commun.* **16**, 785 (2025).

6. A. A. Ordonez, M. A. Sellmyer, G. Gowrishankar, C. A. Ruiz-Bedoya, E. W. Tucker, C. J. Palestro, D. A. Hammoud, S. K. Jain, Molecular imaging of bacterial infections: Overcoming the barriers to clinical translation. *Sci. Transl. Med.* **11**, eaax8251 (2019).

7. L. J. Jara, G. Medina, M. A. Saavedra, Autoimmune manifestations of infections. *Curr. Opin. Rheumatol.* **30**, 373–379 (2018).

8. M. A. Sellmyer, I. Lee, C. Hou, C. C. Weng, S. Li, B. P. Lieberman, C. Zeng, D. A. Mankoff, R. H. Mach, Bacterial infection imaging with [^{18}F]fluoropropyl-trimethoprim. *Proc. Natl. Acad. Sci. U.S.A.* **114**, 8372–8377 (2017).

9. E. C. Lydon, R. Henao, T. W. Burke, M. Aydin, B. P. Nicholson, S. W. Glickman, V. G. Fowler, E. B. Quackenbush, C. B. Cairns, S. F. Kingsmore, A. K. Jaehne, E. P. Rivers, R. J. Langley, E. Petzold, E. R. Ko, M. T. McClain, G. S. Ginsburg, C. W. Woods, E. L. Tsalik,

Validation of a host response test to distinguish bacterial and viral respiratory infection. *EBioMedicine* **48**, 453–461 (2019).

10. Y. Zhou, J. Dong, P. Zhao, J. Zhang, M. Zheng, J. Feng, Imaging of single bacteria with electrochemiluminescence microscopy. *J. Am. Chem. Soc.* **145**, 8947–8953 (2023).

11. J. F. Huggett, D. M. O'Sullivan, S. Cowen, M. H. Cleveland, K. Davies, K. Harris, J. Moran-Gilad, A. Winter, J. Braybrook, M. Messenger, Ensuring accuracy in the development and application of nucleic acid amplification tests (NAATs) for infectious disease. *Mol. Aspects Med.* **97**, 101275 (2024).

12. A. Y. Trick, J. H. Melendez, F.-E. Chen, L. Chen, A. Onzia, A. Zawedde, E. Nakku-Joloba, P. Kyambadde, E. Mande, J. Matovu, M. Atuheirwe, R. Kwizera, E. A. Gilliams, Y.-H. Hsieh, C. A. Gaydos, Y. C. Manabe, M. M. Hamill, T.-H. Wang, A portable magnetofluidic platform for detecting sexually transmitted infections and antimicrobial susceptibility. *Sci. Transl. Med.* **13**, eabf6356 (2021).

13. M. Z. Israr, D. Bernieh, A. Salzano, S. Cassambai, Y. Yazaki, T. Suzuki, Matrix-assisted laser desorption ionisation (MALDI) mass spectrometry (MS): Basics and clinical applications. *Clin. Chem. Lab. Med.* **58**, 883–896 (2020).

14. T. S. Cohen, J. J. Hilliard, O. Jones-Nelson, A. E. Keller, T. O'Day, C. Tkaczyk, A. DiGiandomenico, M. Hamilton, M. Pelletier, Q. Wang, B. A. Diep, V. T. Le, L. Cheng, J. Suzich, C. K. Stover, B. R. Sellman, *Staphylococcus aureus* α toxin potentiates opportunistic bacterial lung infections. *Sci. Transl. Med.* **8**, 329ra331 (2016).

15. S. Niggli, R. Kümmerli, Strain background, species frequency, and environmental conditions are important in determining *Pseudomonas aeruginosa* and *Staphylococcus aureus* population dynamics and species coexistence. *Appl. Environ. Microbiol.* **86**, e00962-20 (2020).

16. O. Jones-Nelson, J. J. Hilliard, A. DiGiandomenico, P. Warrener, A. Alfaro, L. Cheng, C. K. Stover, T. S. Cohen, B. R. Sellman, The neutrophilic response to *Pseudomonas* damages the

airway barrier, promoting infection by *Klebsiella pneumoniae*. *Am. J. Respir. Cell Mol. Biol.* **59**, 745–756 (2018).

17. S. A. Riquelme, D. Ahn, A. Prince, *Pseudomonas aeruginosa* and *Klebsiella pneumoniae* adaptation to innate immune clearance mechanisms in the lung. *J. Innate Immun.* **10**, 442–454 (2018).

18. K. H. Kim, A. Hwang, Y. Song, W. S. Lee, J. Moon, J. Jeong, N. H. Bae, Y. M. Jung, J. Jung, S. Ryu, S. J. Lee, B. G. Choi, T. Kang, K. G. Lee, 3D hierarchical nanotopography for on-site rapid capture and sensitive detection of infectious microbial pathogens. *ACS Nano* **15**, 4777–4788 (2021).

19. J. Dong, X. Wu, Q. Hu, C. Sun, J. Li, P. Song, Y. Su, L. Zhou, An immobilization-free electrochemical biosensor based on CRISPR/Cas13a and FAM-RNA-MB for simultaneous detection of multiple pathogens. *Biosens. Bioelectron.* **241**, 115673 (2023).

20. D. Cai, Y. Wang, J. Zou, Z. Li, E. Huang, X. Ouyang, Z. Que, Y. Luo, Z. Chen, Y. Jiang, G. Zhang, H. Wu, D. Liu, Droplet encoding-pairing enabled multiplexed digital loop-mediated isothermal amplification for simultaneous quantitative detection of multiple pathogens. *Adv. Sci.* **10**, e2205863 (2023).

21. W. Gu, X. Deng, M. Lee, Y. D. Sucu, S. Arevalo, D. Stryke, S. Federman, A. Gopez, K. Reyes, K. Zorn, H. Sample, G. Yu, G. Ishpuniani, B. Briggs, E. D. Chow, A. Berger, M. R. Wilson, C. Wang, E. Hsu, S. Miller, J. L. DeRisi, C. Y. Chiu, Rapid pathogen detection by metagenomic next-generation sequencing of infected body fluids. *Nat. Med.* **27**, 115–124 (2021).

22. Y. K. Cho, H. Kim, A. Bénard, H.-K. Woo, F. Czubayko, P. David, F. J. Hansen, J. I. Lee, J. H. Park, E. Schneck, G. F. Weber, I.-S. Shin, H. Lee, Electrochemiluminescence in paired signal electrode (ECLipse) enables modular and scalable biosensing. *Sci. Adv.* **8**, eabq4022 (2022).

23. S. Wang, S. Zhu, Z. Kang, Y. Chen, X. Liu, Z. Deng, K. Hu, G. Wang, Y. Zhang, G. Zang, Recent advances and future prospects of the potential-resolved strategy in ratiometric,

multiplex, and multicolor electrochemiluminescence analysis. *Theranostics* **12**, 6779–6808 (2022).

24. W. Lv, H. Ye, Z. Yuan, X. Liu, X. Chen, W. Yang, Recent advances in electrochemiluminescence-based simultaneous detection of multiple targets. *TrAC Trends Anal. Chem.* **123**, 115767 (2020).

25. L. Yang, Q. Li, Z. Ge, C. Fan, W. Huang, DNA mechanics: From single stranded to self-assembled. *Nano Lett.* **24**, 11768–11778 (2024).

26. P. Zhan, M. J. Urban, S. Both, X. Duan, A. Kuzyk, T. Weiss, N. Liu, DNA-assembled nanoarchitectures with multiple components in regulated and coordinated motion. *Sci. Adv.* **5**, eaax6023 (2019).

27. S. Chaithongyot, N. Chomanee, K. Charngkaew, A. Udomprasert, T. Kangsamaksin, Aptamer-functionalized DNA nanosphere as a stimuli-responsive nanocarrier. *Mater. Lett.* **214**, 72–75 (2018).

28. Q. Jiang, S. Zhao, J. Liu, L. Song, Z. G. Wang, B. Ding, Rationally designed DNA-based nanocarriers. *Adv. Drug Deliv. Rev.* **147**, 2–21 (2019).

29. Y. Zeng, R. L. Nixon, W. Liu, R. Wang, The applications of functionalized DNA nanostructures in bioimaging and cancer therapy. *Biomaterials* **268**, 120560 (2021).

30. F. Wang, W. Li, X. Feng, D. Liu, Y. Zhang, Decoration of Pt on Cu/Co double-doped CeO(2) nanospheres and their greatly enhanced catalytic activity. *Chem. Sci.* **7**, 1867–1873 (2016).

31. J. H. Lee, D. Y. Jo, J. W. Choung, C. H. Kim, H. C. Ham, K.-Y. Lee, Roles of noble metals (M = Ag, Au, Pd, Pt and Rh) on CeO_2 in enhancing activity toward soot oxidation: Active oxygen species and DFT calculations. *J. Hazard. Mater.* **403**, 124085 (2021).

32. X. Song, L. Zhao, C. Luo, X. Ren, L. Yang, Q. Wei, Peptide-based biosensor with a luminescent copper-based metal-organic framework as an electrochemiluminescence emitter for trypsin assay. *Anal. Chem.* **93**, 9704–9710 (2021).

33. L. Zhao, X. Song, X. Ren, D. Fan, Q. Wei, D. Wu, Rare self-luminous mixed-valence Eu-MOF with a self-enhanced characteristic as a near-infrared fluorescent ECL probe for nondestructive immunodetection. *Anal. Chem.* **93**, 8613–8621 (2021).

34. Y. Zhou, J. He, C. Zhang, J. Li, X. Fu, W. Mao, W. Li, C. Yu, Novel Ce(III)-metal organic framework with a luminescent property to fabricate an electrochemiluminescence immunosensor. *ACS Appl. Mater. Interfaces* **12**, 338–346 (2020).

35. J. J. Nogueira, F. Plasser, L. González, Electronic delocalization, charge transfer and hypochromism in the UV absorption spectrum of polyadenine unravelled by multiscale computations and quantitative wavefunction analysis. *Chem. Sci.* **8**, 5682–5691 (2017).

36. J. Shu, Z. Han, T. Zheng, D. Du, G. Zou, H. Cui, Potential-resolved multicolor electrochemiluminescence of *N*-(4-aminobutyl)-*N*-ethylisoluminol/tetra(4-carboxyphenyl) porphyrin/TiO_2 nanoluminophores. *Anal. Chem.* **89**, 12636–12640 (2017).

37. A. R. Sekhar, Y. Chitose, J. Janoš, S. I. Dangoor, A. Ramundo, R. Satchi-Fainaro, P. Slavíček, P. Klán, R. Weinstain, Porphyrin as a versatile visible-light-activatable organic/metal hybrid photoremovable protecting group. *Nat. Commun.* **13**, 3614 (2022).

38. Z. Yang, S. Luo, Y. Zeng, C. Shi, R. Li, Albumin-mediated biomineralization of shape-controllable and biocompatible ceria nanomaterials. *ACS Appl. Mater. Interfaces* **9**, 6839–6848 (2017).

39. Z. Yang, S. Luo, H. Li, S. Dong, J. He, H. Jiang, R. Li, X. Yang, Alendronate as a robust anchor for ceria nanoparticle surface coating: Facile binding and improved biological properties. *RSC Adv.* **4**, 59965–59969 (2014).

40. J. Shen, X. Zhou, Y. Shan, H. Yue, R. Huang, J. Hu, D. Xing, Sensitive detection of a bacterial pathogen using allosteric probe-initiated catalysis and CRISPR-Cas13a amplification reaction. *Nat. Commun.* **11**, 267 (2020).

41. J. Wu, B. Tang, Y. Qiu, R. Tan, J. Liu, J. Xia, J. Zhang, J. Huang, J. Qu, J. Sun, X. Wang, H. Qu, Clinical validation of a multiplex droplet digital PCR for diagnosing suspected

bloodstream infections in ICU practice: A promising diagnostic tool. *Crit. Care* **26**, 243 (2022).

42. H. Cheng, P. Hui, J. Peng, W. Li, W. Ma, H. Wang, J. Huang, X. He, K. Wang, Enzymatic behavior regulation-based colorimetric and electrochemiluminescence sensing of phosphate using the cobalt oxyhydroxide nanosheet. *Anal. Chem.* **93**, 6770–6778 (2021).

43. Y. Li, Y. Wang, Q. Wu, R. Qi, L. Li, L. Xu, H. Yuan, High-throughput fluorescence sensing array based on tetraphenylethylene derivatives for detecting and distinguishing pathogenic microbes. *Spectrochim. Acta A Mol. Biomol. Spectrosc.* **318**, 124435 (2024).

44. E. L. Lawrence, B. D. A. Levin, T. Boland, S. L. Y. Chang, P. A. Crozier, Atomic scale characterization of fluxional cation behavior on nanoparticle surfaces: Probing oxygen vacancy creation/annihilation at surface sites. *ACS Nano* **15**, 2624–2634 (2021).

45. V. Baldim, F. Bedioui, N. Mignet, I. Margaill, J. F. Berret, The enzyme-like catalytic activity of cerium oxide nanoparticles and its dependency on Ce(3+) surface area concentration. *Nanoscale* **10**, 6971–6980 (2018).

46. H. Dong, L. Zhang, L. Li, W. Deng, C. Hu, Z.-J. Zhao, J. Gong, Abundant Ce^{3+} ions in Au-CeO_x nanosheets to enhance CO_2 electroreduction performance. *Small* **15**, e1900289 (2019).

47. N. Balhara, M. Devi, A. Balda, M. Phour, A. Giri, Urine; a new promising biological fluid to act as a non-invasive biomarker for different human diseases. *URINE* **5**, 40–52 (2023).

48. A. Nath, S. M. Larsson, A. Lenshof, W. Qiu, T. Baasch, L. Nilsson, M. Gram, D. Ley, T. Laurell, Acoustophoresis-based blood sampling and plasma separation for potentially minimizing sampling-related blood loss. *Clin. Chem. Lab. Med.* **63**, 2218–2225 (2025).

49. L. M. Kopcinovic, J. Culej, Pleural, peritoneal and pericardial effusions – A biochemical approach. *Biochem. Med.* **24**, 123–137 (2014).

50. T. T.-Q. Nguyen, E. R. Kim, M. B. Gu, A new cognate aptamer pair-based sandwich-type electrochemical biosensor for sensitive detection of *Staphylococcus aureus*. *Biosens. Bioelectron.* **198**, 113835 (2022).

51. X. Cao, S. Li, L. Chen, H. Ding, H. Xu, Y. Huang, J. Li, N. Liu, W. Cao, Y. Zhu, B. Shen, N. Shao, Combining use of a panel of ssDNA aptamers in the detection of *Staphylococcus aureus*. *Nucleic Acids Res.* **37**, 4621–4628 (2009).

52. R. Li, J. Yan, B. Feng, M. Sun, C. Ding, H. Shen, J. Zhu, S. Yu, Ultrasensitive detection of multidrug-resistant bacteria based on boric acid-functionalized fluorescent MOF@COF. *ACS Appl. Mater. Interfaces* **15**, 18663–18671 (2023).

53. Y. Sun, X. Cheng, Y. Yi, K. Quan, Q. Chen, K. Zhang, J. J. Xu, The compact integration of multiple exonuclease III-assisted cyclic amplification units for high-efficiency ratiometric electrochemiluminescence detection of MRSA. *Anal. Chem.* **96**, 943–948 (2024).

54. I. Ocsoy, S. Yusufbeyoglu, V. Yılmaz, E. S. McLamore, N. Ildız, A. Ülgen, DNA aptamer functionalized gold nanostructures for molecular recognition and photothermal inactivation of methicillin-Resistant *Staphylococcus aureus*. *Colloids Surf. B Biointerfaces* **159**, 16–22 (2017).

55. J. Soundy, D. Day, Selection of DNA aptamers specific for live *Pseudomonas aeruginosa*. *PLOS ONE* **12**, e0185385 (2017).

56. J. Hu, K. Fu, P. W. Bohn, Whole-cell *Pseudomonas aeruginosa* localized surface plasmon resonance aptasensor. *Anal. Chem.* **90**, 2326–2332 (2018).

57. X. Shi, J. Zhang, F. He, A new aptamer/polyadenylated DNA interdigitated gold electrode piezoelectric sensor for rapid detection of *Pseudomonas aeruginosa*. *Biosens. Bioelectron.* **132**, 224–229 (2019).

58. Z. Zhong, R. Gao, Q. Chen, L. Jia, Dual-aptamers labeled polydopamine-polyethyleneimine copolymer dots assisted engineering a fluorescence biosensor for sensitive detection of *Pseudomonas aeruginosa* in food samples. *Spectrochim. Acta A Mol. Biomol. Spectrosc.* **224**, 117417 (2020).

59. C. Y. Effah, L. Ding, L. Tan, S. He, X. Li, H. Yuan, Y. Li, S. Liu, T. Sun, Y. Wu, A SERS bioassay based on vancomycin-modified PEI-interlayered nanocomposite and aptamer-

functionalized SERS tags for synchronous detection of *Acinetobacter baumannii* and *Klebsiella pneumoniae*. *Food Chem.* **423**, 136242 (2023).

60. A. Deb, M. Gogoi, T. K. Mandal, S. Sinha, P. S. G. Pattader, Specific instantaneous detection of *Klebsiella pneumoniae* for UTI diagnosis with a plasmonic gold nanoparticle conjugated aptasensor. *ACS Appl. Bio. Mater.* **6**, 3309–3318 (2023).

61. S. Liu, Q. Li, H. Yang, P. Wang, X. Miao, Q. Feng, An in situ quenching electrochemiluminescence biosensor amplified with aptamer recognition-induced multi-DNA release for sensitive detection of pathogenic bacteria. *Biosens. Bioelectron.* **196**, 113744 (2022).

62. R. Das, A. Dhiman, A. Kapil, V. Bansal, T. K. Sharma, Aptamer-mediated colorimetric and electrochemical detection of *Pseudomonas aeruginosa* utilizing peroxidase-mimic activity of gold NanoZyme. *Anal. Bioanal. Chem.* **411**, 1229–1238 (2019).

63. S. Meile, J. Du, S. Staubli, S. Grossmann, H. Koliwer-Brandl, P. Piffaretti, L. Leitner, C. I. Matter, J. Baggenstos, L. Hunold, S. Milek, C. Guebeli, M. Kozomara-Hocke, V. Neumeier, A. Botteon, J. Klumpp, J. Marschall, S. McCallin, R. Zbinden, T. M. Kessler, M. J. Loessner, M. Dunne, S. Kilcher, Engineered reporter phages for detection of *Escherichia coli*, *Enterococcus*, and *Klebsiella* in urine. *Nat. Commun.* **14**, 4336 (2023).

64. H. Peng, I. A. Chen, Rapid colorimetric detection of bacterial species through the capture of gold nanoparticles by chimeric phages. *ACS Nano* **13**, 1244–1252 (2019).

65. M. Imai, K. Mine, H. Tomonari, J. Uchiyama, S. Matuzaki, Y. Niko, S. Hadano, S. Watanabe, Dark-field microscopic detection of bacteria using bacteriophage-immobilized SiO_2@AuNP core-shell nanoparticles. *Anal. Chem.* **91**, 12352–12357 (2019).

66. P. Liu, Y. Wang, L. Han, Y. Cai, H. Ren, T. Ma, X. Li, V. A. Petrenko, A. Liu, Colorimetric assay of bacterial pathogens based on Co_3O_4 magnetic nanozymes conjugated with specific fusion phage proteins and magnetophoretic chromatography. *ACS Appl. Mater. Interfaces* **12**, 9090–9097 (2020).

67. B. Duncan, N. D. B. Le, C. Alexander, A. Gupta, G. Y. Tonga, M. Yazdani, R. F. Landis, L.-S. Wang, B. Yan, S. Burmaoglu, X. Li, V. M. Rotello, Sensing by smell: Nanoparticle-enzyme sensors for rapid and sensitive detection of bacteria with olfactory output. *ACS Nano* **11**, 5339–5343 (2017).

68. D. Svechkarev, M. R. Sadykov, K. W. Bayles, A. M. Mohs, Ratiometric fluorescent sensor array as a versatile tool for bacterial pathogen identification and analysis. *ACS Sens.* **3**, 700–708 (2018).

69. A. Suea-Ngam, P. D. Howes, A. J. deMello, An amplification-free ultra-sensitive electrochemical CRISPR/Cas biosensor for drug-resistant bacteria detection. *Chem. Sci.* **12**, 12733–12743 (2021).

70. J. Li, W. Shen, X. Liang, S. Zheng, Q. Yu, C. Wang, C. Wang, B. Gu, 2D film-like magnetic SERS tag with enhanced capture and detection abilities for immunochromatographic diagnosis of multiple bacteria. *Small* **20**, e2310014 (2024).

71. J. Li, Z. Li, B. Wang, Q. Yu, T. Wu, C. Wang, B. Gu, Electropositive magnetic fluorescent nanoprobe-mediated immunochromatographic assay for the ultrasensitive and simultaneous detection of bacteria. *Adv. Sci.* **12**, e2412421 (2025).

72. E. A. Idelevich, U. Reischl, K. Becker, New microbiological techniques in the diagnosis of bloodstream infections. *Dtsch. Arztebl. Int.* **115**, 822–832 (2018).

73. G. Jannes, D. De Vos, in *Diagnostic Bacteriology Protocols*, L. O'Connor, Ed. (Humana Press, 2006), pp. 1–21.

74. D. Wang, S. Wang, X. Du, Q. He, Y. Liu, Z. Wang, K. Feng, Y. Li, Y. Deng, ddPCR surpasses classical qPCR technology in quantitating bacteria and fungi in the environment. *Mol. Ecol. Resour.* **22**, 2587–2598 (2022).

75. M. J. Espy, J. R. Uhl, L. M. Sloan, S. P. Buckwalter, M. F. Jones, E. A. Vetter, J. D. C. Yao, N. L. Wengenack, J. E. Rosenblatt, F. R. Cockerill III, T. F. Smith, Real-time PCR in clinical microbiology: Applications for routine laboratory testing. *Clin. Microbiol. Rev.* **19**, 165–256 (2006).

76. Y. Peng, R. Xie, Y. Luo, P. Guo, Z. Wu, Y. Chen, P. Liu, J. Deng, B. Huang, K. Liao, Clinical evaluation of a multiplex droplet digital PCR for diagnosing suspected bloodstream infections: A prospective study. *Front. Cell. Infect. Microbiol.* **14**, 1489792 (2024).

77. A. van Belkum, M. Welker, M. Erhard, S. Chatellier, Biomedical mass spectrometry in today's and tomorrow's clinical microbiology laboratories. *J. Clin. Microbiol.* **50**, 1513–1517 (2012).
